# Socio-affective communication in *Tph2*-deficient rat pups: communal nesting aggravates growth retardation despite ameliorating maternal affiliation deficits

**DOI:** 10.1186/s13229-024-00629-x

**Published:** 2024-11-29

**Authors:** Tianhua Wang, Judith R. Homberg, Laura Boreggio, Marta C. F. Samina, Rogério C. R. Castro, Sharon M. Kolk, Natalia Alenina, Michael Bader, Jinye Dai, Markus Wöhr

**Affiliations:** 1 Faculty of Psychology, Experimental and Biological Psychology, Philipps-Universität Marburg, Behavioral Neuroscience, 35032 Marburg, Germany; 2 Philipps-Universität Marburg, Center for Mind, Brain, and Behavior (CMBB), 35032 Marburg, Germany; 3 Department of Cognitive Neuroscience, Radboud University Medical Center, Donders Institute for Brain, Cognition, and Behaviour, 6525 EN Nijmegen, The Netherlands; 4 Molecular Biology of Peptide Hormones, Max-Delbrück-Centrum Für Molekulare Medizin (MDC), 13125 Berlin, Germany; 5 Radboud University, Donders Institute for Brain, Cognition, and Behaviour, 6525 AJ Nijmegen, The Netherlands; 6 German Center for Cardiovascular Research (DZHK), partner site Berlin, 10785 Berlin, Germany; 7 Charité University Medicine Berlin, 10117 Berlin, Germany; 8 Institute for Biology, University of Lübeck, 23562 Lübeck, Germany; 9 Department of Pharmacological Sciences and Department of Neuroscience, Mount Sinai, Icahn School of Medicine, Friedman Brain Institute, New York, 10029 USA; 10 Faculty of Psychology and Educational Sciences, Research Unit Brain and Cognition, Laboratory of Biological Psychology, Social and Affective Neuroscience Research Group, KU Leuven, Tiensestraat 102 - Bus 3714, 3000 Louvain, Belgium; 11 KU Leuven, Leuven Brain Institute, 3000 Louvain, Belgium

**Keywords:** Serotonin, Tryptophan hydroxylase 2, Autism, Neurodevelopmental disorders, Social behavior, Ultrasonic vocalizations

## Abstract

**Background:**

A lack of serotonin (also known as 5-hydroxytryptamine, 5-HT) in the brain due to deficiency of the rate-limiting enzyme in 5-HT synthesis, tryptophan hydroxylase 2 (TPH2), was recently reported to result in impaired maternal affiliation across species, including mice, rats, and monkeys. In rodents, this was reflected in a lack of preference for maternal odors and reduced levels of isolation-induced ultrasonic vocalizations (USV), possibly contributing to a severe growth retardation phenotype.

**Methods:**

Here, we tested whether growth retardation, maternal affiliation deficits, and/or impairments in socio-affective communication caused by *Tph2* deficiency can be rescued through early social enrichment in rats. To this aim, we compared male and female *Tph2*^*−/−*^ knockout and *Tph2*^*+/−*^ heterozygous rat pups to *Tph2*^*+/+*^ wildtype littermate controls, with litters being randomly assigned to standard nesting (SN; one mother with her litter) or communal nesting (CN; two mothers with their two litters).

**Results:**

Our results show that *Tph2* deficiency causes severe growth retardation, together with moderate impairments in somatosensory reflexes and thermoregulatory capabilities, partially aggravated by CN. *Tph2* deficiency further led to deficits in socio-affective communication, as evidenced by reduced emission of isolation-induced USV, associated with changes in acoustic features, clustering of subtypes, and temporal organization. Although CN did not rescue the impairments in socio-affective communication, CN ameliorated the maternal affiliation deficit caused by *Tph2* deficiency in the homing test. To close the communicative loop between mother and pup, we assessed maternal preference and showed that mothers display a preference for *Tph2*^*+/+*^ controls over *Tph2*^*−/−*^ pups, particularly under CN conditions. This is consistent with the aggravated growth phenotype in *Tph2*^*−/−*^ pups exposed to the more competitive CN environment.

**Conclusion:**

Together, this indicates that CN aggravates growth retardation despite ameliorating maternal affiliation deficits in *Tph2*-deficient rat pups, possibly due to reduced and acoustically altered isolation-induced USV, hindering efficient socio-affective communication between mother and pup.

**Supplementary Information:**

The online version contains supplementary material available at 10.1186/s13229-024-00629-x.

## Introduction

Serotonin (also known as 5-hydroxytryptamine, 5-HT) is a monoamine neurotransmitter with complex biological functions. As neuromodulator it regulates neuronal signaling involved in a broad variety of psychological processes and functions, ranging from motor control and appetite to mood and social behavior [45]. Consequently, the 5-HT system is strongly involved in mediating the effects of a diverse group of psychoactive drugs with anxiolytic, antidepressant, neuroleptic, empathogenic, and psychedelic properties [57]. However, 5-HT likewise plays a prominent role in regulating various aspects of neurodevelopment [31]. This includes cell proliferation, differentiation, and migration as well as network formation, e.g. through controlling synaptogenesis [26]. In line with its function during development, the 5-HT system is one of the earliest developing neurotransmitter systems. In fact, the expression of 5-HT together with associated enzymes and receptors peaks during early brain development and alterations therein have been associated with neurodevelopmental disorders [56], such as autism spectrum disorder (ASD; [10]).

A key element of the central 5-HT system is the enzyme tryptophan hydroxylase 2 (TPH2), the initial and rate-limiting factor in 5-HT synthesis in the brain [76]. Removing TPH2 from experimental animals through genetic engineering was found to result in altered neurodevelopmental profiles in mice, rats, pigs, and monkeys. One of the most prominent effects of congenital lack of brain 5-HT is growth retardation associated with low survival rates during early development [2]. In mice, growth retardation was associated with reduced brain growth and delayed cortical maturation [59]. Growth retardation and low survival rates were also reported in *Tph2*-deficient rats [37] and pigs [44], while no prominent phenotype was reported in monkeys [47].

At the behavioral level, *Tph2* deficiency was found to cause a multitude of behavioral alterations. In mice, this includes decreased levels of anxiety-related behavior [7, 55], while panic-like escape responses and fear learning appear to be facilitated [74, 75]. Assessment of depression-like behavior led to variable results [6, 50, 55, 65]. In rats, lower levels of anxiety-related behavior were reported [52].

A behavioral domain particularly affected by *Tph2* deficiency is social behavior. This includes maternal caregiving behavior and evidence for maternal neglect was repeatedly observed in independent mouse studies [2, 8, 58]. Moreover, altered social interaction behavior was seen in juvenile and adult mice [9], possibly due to reduced social interest and social memory deficits [38]. Particularly in adult mice, strongly enhanced levels of aggressive behavior were evident, both in males and females [2, 27, 39, 50, 55], possibly due to an increase in impulsivity [7] and potentiated by social isolation [48]. This includes increased levels of mounting behavior between males, which was interpreted as evidence for a lack of sexual preference [46], also reported in females [88],but see: [5, 9]. Increased aggressive behavior and changes in sexual behavior were also seen in rats [4, 52, 62].

An important component of the rich social behavior repertoire of rodents is socio-affective communication through ultrasonic vocalizations (USV; [15, 63, 86]). Mice and rats emit distinct types of USV. During the first two weeks of life, mouse and rat pups emit isolation-induced USV when separated from mother and littermates [3, 90]. Such isolation-induced USV serve important communicative functions in regulating mother–pup interactions and were shown to stimulate maternal caregiving behavior [71, 85]. In juvenile and adult mice, ultrasonic calling is prominent during affiliative social interactions [61]. High rates are also seen in adult males exposed to female urine [64], presumably to attract females [28].

Across species, congenital deficiency in brain 5-HT due to *Tph2* deletion has been implicated in the regulation of socio-affective communication. In mice, *Tph2* deficiency was found to result in a prominent deficit in the emission of pup isolation-induced USV, as reflected in lower call emission rates, altered call clustering, and reduced temporal organization [54]. Moreover, ultrasonic calling in response to female urine was virtually absent in adult *Tph2*-deficient male mice [9]. Importantly, socio-affective communication deficits in mouse pups were recently linked to reduced maternal affiliation, as reflected in a lack of preference for maternal odors [47]. Moreover, similar deficits in socio-affective communication and maternal affiliation were evident in rats and monkeys, suggesting a conserved function of 5-HT in regulating maternal preference and pup-driven interactions with the mother [47].

A central element of the early environment shaping the behavioral repertoire of the offspring is maternal care and the interaction with littermates. In the laboratory, communal rearing or nesting are often used to systematically study the impact of those early social environmental factors [12]. Communal rearing or nesting, e.g. two mothers and their litters, as opposed to standard conditions with one mother and her litter, was shown to promote the acquisition of social skills [13] and resilience to stress [14].

Here, we tested whether growth retardation, maternal affiliation deficits, and/or impairments in socio-affective communication caused by *Tph2* deficiency can be rescued through early social enrichment in rats. To this aim, we compared male and female *Tph2*^*−/−*^ knockout and *Tph2*^*+/−*^ heterozygous rat pups to *Tph2*^*+/+*^ wildtype littermate controls, with litters being randomly assigned to standard nesting (SN; one mother with her litter) or communal nesting (CN; two mothers with their two litters). This was followed by the assessment of socio-affective communication through isolation-induced USV and the homing test as a proxy for maternal affiliation, in combination with the assessment of developmental milestones, somatosensory reflexes, and thermoregulatory capabilities in the rat pups throughout the first two weeks of life. Importantly, our approach included not only the sender emitting isolation-induced USV but also the receiver responding to isolation-induced USV through including a maternal preference test, to truly model the reciprocal nature of communication.

## Methods

### Animals and housing

Effects of early social enrichment on growth retardation, maternal affiliation deficits, and impairments in socio-affective communication were assessed in *Tph2*^*−/−*^ knockout (KO) and *Tph2*^*+/−*^ heterozygous (HET) rat pups and compared to *Tph2*^*+/+*^ wildtype (WT) littermate controls, with balanced representation of sexes. *Tph2*^*+/−*^ rats carrying a 10-base pair deletion in exon 7 were generated by means of zinc finger technology on a Dark Agouti background [37]. *Tph2*^*+/−*^ founders were provided by the Max-Delbrück-Centrum für Molekulare Medizin, Germany [67], and backcrossed after arrival at Philipps-Universität Marburg, Germany, by pairing them with Dark Agouti rats (DA/OlaHsd, Janvier, France).

**Fig. 1:**
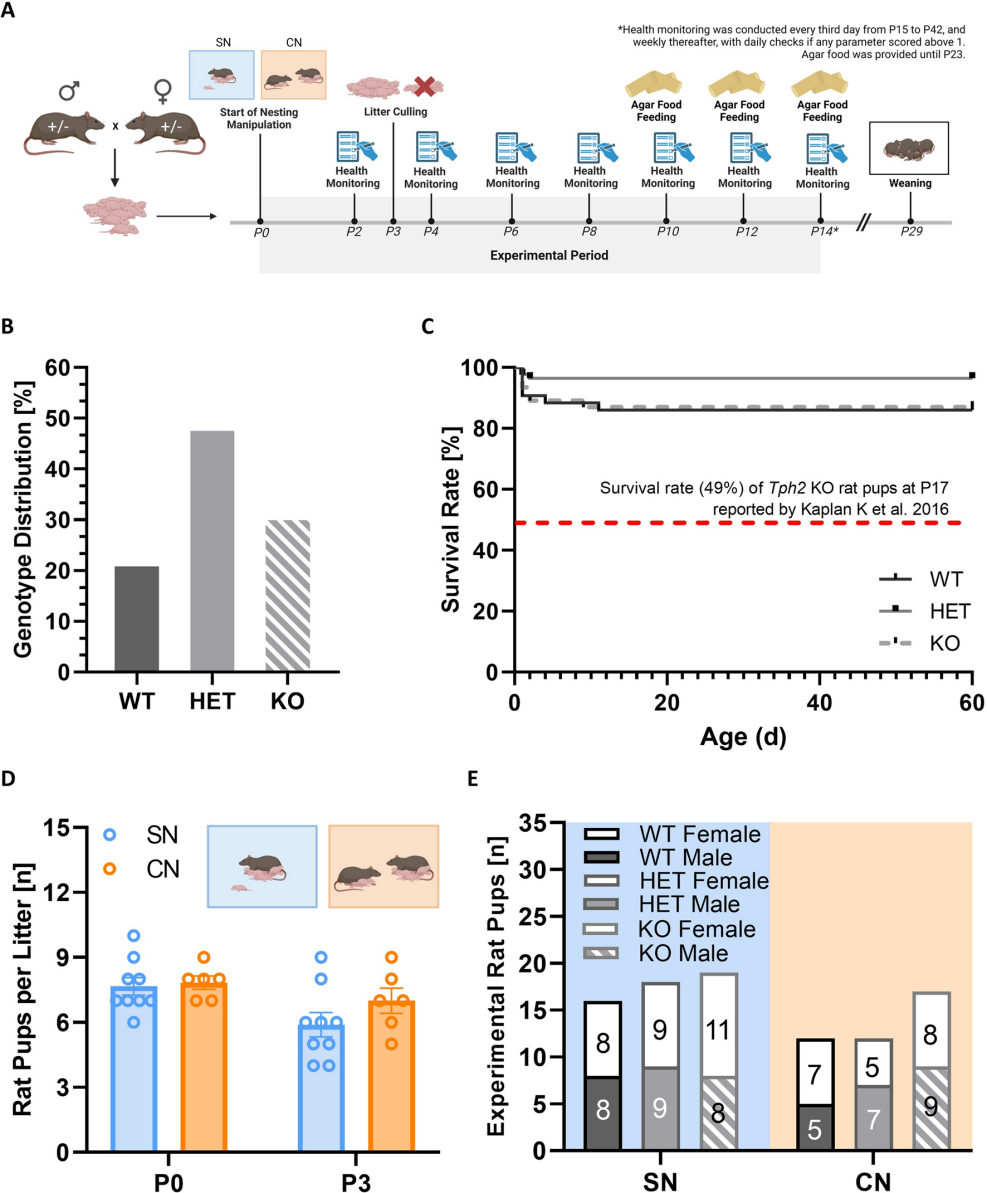
Overview of experimental design, genotype distribution, and survival rates. (**A**) A heterozygous breeding strategy was applied to obtain male and female *Tph2*^*−/−*^ knockout and *Tph2*^*+/−*^ heterozygous rat pups, together with *Tph2*^*+/+*^ wildtype littermate controls. Litters were randomly assigned to standard nesting (SN; one mother with her litter) or communal nesting (CN; two mothers with their two litters). An intensive health monitoring and care system was applied to mitigate for the low survival rates previously reported to be associated with *Tph2* deficiency in mice and rats. (**B**) Genotype distribution of rat pups obtained through applying a heterozygous breeding strategy, including all N = 33 litters obtained. (**C**) Survival rate of *Tph2*^*−/−*^ (KO) and *Tph2*^*+/−*^ (HET) rat pups, as compared to *Tph2*^*+/+*^ (WT) littermate controls; including all N = 33 litters obtained, with rat pups removed through culling from the litter to mitigate for the low survival rates not included in calculating survival rates as percentages. (**D**) Litter sizes of the N = 15 experimental litters from SN and CN included in the analyses on postnatal day 0 (P0) and postnatal day 3 (P3). (**E**) Genotype distribution of experimental rat pups included in the analyses, split according to sex

For experiments, a heterozygous breeding strategy was applied to obtain *Tph2*^*−/−*^ and *Tph2*^*+/−*^ offspring and *Tph2*^*+/+*^ littermate controls (Fig. 1A). The day of birth was defined as postnatal day (P) 0. Rats were housed in standard Makrolon Type IV cages with high stainless-steel covers (59 × 33 × 20 cm) under standard laboratory conditions. Cages were kept in temperature- and humidity-controlled rooms (22 ± 2 °C, 40–70%) with a 12:12 h light/dark cycle (lights on at 7:00 am). Standard rodent chow (Altromin, Lage, Germany) and water were available ad libitum. To mitigate for the low survival rates reported in *Tph2*^*−/−*^ rats before [37], litter size was reduced after genotyping on P2 by randomly culling over-represented genotypes for large litters on P3, typically aiming for N = 1 rat pup per sex and genotype. Moreover, agar food (Solid Drink®-DeHyPrev Bio 75 gm, SDDHP-75, 3at-bio b.v., Tiel, The Netherlands) was provided from P10 to increase survival rates. Health monitoring was performed on P2, P4, P6, P8, P10, P12, and P14. Heterozygous breeding resulted in N = 33 litters with a genotype distribution of approximately 30% *Tph2*^*−/−*^, 50% *Tph2*^*+/−*^ offspring, and 20% *Tph2*^*+/+*^ littermate controls (Fig. 1B). Measures taken to increase survival rates were successful. As compared to a previously reported survival rate of approximately 50% for *Tph2*^*−/−*^ rat pups [37], a much higher survival rate of approximately 90% was obtained for *Tph2*^*−/−*^ offspring, reaching levels comparable to *Tph2*^*+/−*^ offspring and *Tph2*^*+/+*^ littermate controls (Fig. 1C). To avoid litter effects, only litters with *Tph2*^*+/+*^ littermate controls and *Tph2*^*+/−*^ and/or *Tph2*^*−/−*^ rat pups were included in the experiments, with both sexes present. Of note, for CN litters this rule was applied independently for the two litters housed together. This resulted in the inclusion of N = 15 out of N = 33 litters in the experiments/analyses, of which N = 9 litters were used for SN and N = 6 litters were used for CN. On P0, average litter size was 7.67 ± 0.41 pups for SN and 7.83 ± 0.31 for CN. After culling on P3, average litter size was 5.89 ± 0.56 for SN and 7.00 ± 0.58 for CN (Fig. 1D). The total number of pups included was N = 94, with balanced sex and genotype representations (Fig. 1E).

All procedures were conducted in strict compliance with the National Institutes of Health Guidelines for the Care and Use of Laboratory Animals, the European regulations for animal experiments (2010/63/EU), and the legal requirements of Germany. Procedures were approved by the local authorities (G14/2022).

### Experimental design

To study the effects of early social enrichment on growth retardation, maternal affiliation deficits, and impairments in socio-affective communication in a genotype- and sex-dependent manner, an experimental design with four independent variables was used, namely sex, genotype, developmental stage, and nesting condition, i.e. SN versus CN. To this aim, litters with male and female *Tph2*^*−/−*^, *Tph2*^*+/−*^, and *Tph2*^*+/+*^ littermates were randomly assigned to SN (one mother with her litter) or CN (two mothers with their two litters) from P0 (a metal cage divider with holes was used from P0 to P2 to keep the pups from the two litters separate before tattooing for identification was completed). This was followed by the assessment of developmental milestones, somatosensory reflexes, and thermoregulatory capabilities, together with the recording of isolation-induced USV on P2, P4, P6, P8, P10, P12, and P14 during a 10-min social isolation period. In addition, a maternal preference test was performed on P7 and the homing test as a proxy for maternal affiliation was conducted on P11. Of note, one *Tph2*^*+/+*^ littermate control exposed to CN was injured while the two females present in the cage were competing for it at P10. It was excluded from all data analyses, except the maternal preference test conducted at P7. After completion of behavioral experiments on P2, tail samples were taken for genotyping. Pups were tattooed for their identification using a non-toxic animal tattoo ink (Ketchum permanent tattoo inks green paste, Ketchum Manufacturing Inc., Brockville, Canada). The ink was inserted subcutaneously through a 27-gauge hypodermic needle tip into the center of the paw. All behavioral tests were conducted during the light phase of the 12:12 h light/dark cycle. Experimenters were blind to genotypes during data acquisition and analysis. Prior to each test, the behavioral equipment was cleaned using a 0.1% acetic acid solution and dried with paper towels.

### Genotyping

Genotyping was performed using DNA obtained from tail samples. After the behavioral experiments on P2, approximately 0.3 cm of tails were collected and digested using DirectPCR Lysis Reagent (Viagen, Canada) and proteinase K (PanReac AppliChem, Germany) to obtain crude DNA extracts. Then, 1 μl of the DNA extract was amplified by PCR using the GE Healthcare Illustra™ PuReTaq RTG PCR Bead Kit (Little Chalfont, UK), with the following primers: 5′-ACCTGAGCCCAAGAGACTTCC-3′ (forward) and 5′-GCTACGCTATCAAAGGCCCG-3′ (reverse). The thermocycler (MyCycler, BioRad, Hercules, CA, USA) was programmed for 1 cycle at 94 °C for 180 s, followed by 35 cycles of 94 °C for 30 s, 61 °C for 30 s, and 68 °C for 30 s, and a final cycle at 68 °C for 300 s, to accomplish denaturing, annealing, and extension steps for PCR products. 10 μl of the PCR products were then digested by incubation at 37 °C for 90 min using restriction enzyme Mnl1 and rCutSmart Buffer (NEW ENGLAND Biolabs, Germany). Electrophoresis of the digested PCR products was performed using the Sub-Cell GT system (BioRad, Hercules, CA, USA) with a 3% agarose gel stained with GelRed™ 3X (Biotium, Hayward, CA, USA). Automated gel documentation was conducted using the GelDoc™ imaging system with a UV tray (BioRad, Hercules, CA, USA). Genotyping was completed by P3, after which litter culling was conducted as necessary based on genotyping results.

### Developmental milestones, somatosensory reflexes, and thermoregulatory capabilities

Developmental milestones, somatosensory reflexes, and thermoregulatory capabilities were assessed by a trained experimenter on P2, P4, P6, P8, P10, P12, and P14. First, body temperature was assessed using a thermal camera (testo 865 s, Testo SE & Co. KGaA, Lenzkirch). Body temperature was assessed twice under room temperature in individual rat pups placed on a flat surface, i.e. immediately after removing them from the litter (before isolation) and after the 10-min isolation period (after isolation). Then, after the 10-min social isolation period, body weight and length were measured. Body weight was determined by a laboratory balance (IoT-Line Compact Laboratory Balance PCB 2000–1; KERN & SOHN GmbH, Germany). Next, physical landmarks and somatosensory reflexes were determined, as described in Wöhr et al. [84]. The following physical landmarks were scored: Pinnae detachment, eye opening, incisor eruption, fur development, and milk spot. Somatosensory reflexes were scored in the following order:

1. Grasping reflex: The pup's forepaws were stroked with a toothpick. Grasping the shaft of the toothpick was recorded as present or absent.
2. Forelimb placing: The pup's dorsa of the forepaws were stroked with a toothpick. Raising and placing the forepaw on the toothpick was recorded as present or absent.
3. Righting reflex: The pup was gently held on its back and released. Latency to flip over onto the abdomen with four paws touching the surface was measured with a stopwatch.
4. Level screen: The pup was gently dragged across a square grid (8 × 11 cm) by the tail. Grasping was recorded as present or absent.
5. Negative geotaxis: The pup was gently placed head down on a square of grid (8 × 11 cm) at an angle of 45°. Latency to turn 180° to either side was measured with a stopwatch.
6. Vertical screen: The pup was placed on a square grid (8 × 11 cm) at 90° angle. Duration the pup was able to stay on the grid was measured with a stopwatch.
7. Cliff avoidance: The pup was placed on a rectangular object with flat surface and the pup's snout and forepaws were gently pushed over its edge. Latency to withdraw from the edge was measured with a stopwatch.
8. Bar holding: The pup grasping a small elevated wire bar by its forelimbs, while the hindlimbs were not in contact with the surface. Duration the pup was able to hold onto the bar (including assistance of hindlimbs and tail) was measured with a stopwatch.
9. Auditory startle: The pup was exposed to an acoustic stimulus (hand clapping). Startle response was recorded as present or absent.

Latencies or durations were measured in seconds for righting reflex (maximum: 60 s), negative geotaxis (maximum: 60 s), vertical screen (maximum: 60 s), cliff avoidance (maximum: 30 s), and bar holding (maximum: 60 s). Other somatic and behavioral variables were rated semi-quantitatively: 0 = absent, 1 = uncertain, 2 = present, and 3 = evident.

### Isolation-induced ultrasonic vocalizations: recording

For eliciting isolation-induced USV, pups were separated from mother and littermates and isolated for a period of 10 min under room temperature (ca. 20–23 °C), as previously described [85]. Pups were individually removed from the nest at random and gently placed into an isolation box (23 × 28 × 18 cm) made of white and transparent plastic walls. The roof and one wall were made of transparent plastic to allow video observation during the test. The isolation box was placed in a sound attenuating isolation cubicle (51 × 71 × 51 cm, Coulbourn Instruments, Allentown, PA) equipped with 2 white-light LED spots (63 lx, Conrad Electronic GmbH, Hirschau, Germany) and a black/white CCD camera (Conrad Electronic GmbH) for video observation. For broad-band, high-resolution ultrasound recording of isolation-induced USV, an UltraSoundGate Condenser Microphone CM 16 (Avisoft Bioacoustics, Berlin, Germany) was placed in the roof of the sound attenuating box, 12 cm above the floor. The microphone was connected via an UltraSoundGate 416 USGH audio device (Avisoft Bioacoustics) to a personal computer, where acoustic data were recorded with a sampling rate of 250,000 Hz in 16-bit format by Avisoft RECORDER (version 2.97; Avisoft Bioacoustics). The microphone that was used for recording was sensitive to frequencies of 15 to 180 kHz with a flat frequency response (± 6 dB) between 25 and 140 kHz.

### Isolation-induced ultrasonic vocalizations: analysis

DeepSqueak (DS; version 3.1.0; [18]) with a convolutional neural network (CNN) was used to analyze the isolation-induced USV emitted during the 10-min social isolation period. First, the DS object detector using a faster regional CNN (FASTER-RCNN) was custom-trained without default CNN using over 16,000 images of isolation-induced USV obtained through broad-band, high-resolution ultrasound recording of isolation-induced USV, including images from all developmental stages, i.e. P2-P14. Then, all broad-band, high-resolution ultrasound recordings were processed using the custom-trained FASTER-RCNN, isolation-induced USV were detected, and detection files were generated. The accuracy of call detection by DS was verified manually by an experienced user. When necessary, false positives were eliminated and missed isolation-induced USV were included. False positives were seen rarely, while missings occurred more often, with an estimated number of about 5% of isolation-induced USV on average being added manually. The total number of isolation-induced USV was calculated for the entire 10-min isolation period for all developmental stages, i.e. P2-P14. Besides call number, total calling time and various acoustic features were assessed. For every single isolation-induced USV, the acoustic features determined included call duration, peak frequency, peak amplitude, and frequency modulation. They were calculated for each individual isolation-induced USV separately, i.e. call length (s), principal frequency (kHz), mean power (dB/Hz), and delta frequency (kHz), as measured and defined by DS (for details, please see Fig. 4A). Total calling time was defined as the sum of the lengths of all individual isolation-induced USV. In addition, call subtypes were determined by means of density blots using more than 150,000 isolation-induced USV emitted on P12, applying a data processing approach established before [80]. Specifically, two types of density blots were used, i.e. one depicting peak frequency versus call duration and another one depicting peak frequency versus frequency modulation. Finally, to assess the temporal organization of isolation-induced USV emission, sequential analyses were performed by correlating the call durations of given isolation-induced USV with the call durations of the previous ones (N-1), the ones two before (N-2), and the ones three before (N-3), as applied repeatedly as a proxy for the level of randomness versus stereotypy in the temporal USV emission patterns [80].

### Homing test as a proxy for maternal affiliation

As a proxy for maternal affiliation, the homing test was performed on P11 under dim red-light conditions (room temperature: ca. 20–21 °C). First, pups were individually isolated in a standard Makrolon Type III cage (43 × 27 × 15 cm) containing both soiled bedding from the home cage and clean bedding. The soiled bedding was evenly spread over 1/3 of the cage floor on one side, while the rest was covered with clean bedding. The soiled bedding zone was counter-balanced across tests to avoid positional bias. At the beginning of the test, the pup was placed in the center of the cage floor. Then, the pup was allowed to freely explore the entire cage for 10 min. After the homing test, the pup’s body temperature was measured using a thermal camera (testo 865 s, Testo SE & Co. KGaA, Lenzkirch). For behavior scoring, video recording was performed (IP camera RLC-410-5MP; Reolink, Hong Kong, China). For broad-band, high-resolution ultrasound recording of isolation-induced USV, an UltraSoundGate Condenser Microphone CM 16 (Avisoft Bioacoustics, Berlin, Germany) was positioned 24 cm above the cage floor. The microphone was connected via an UltraSoundGate 416 USGH audio device (Avisoft Bioacoustics) to a personal computer, where acoustic data were recorded with a sampling rate of 250,000 Hz in 16-bit format by Avisoft RECORDER (version 2.97; Avisoft Bioacoustics). To synchronize video and ultrasound recordings for later analysis, a timer was beeped under the camera and microphone. For behavior scoring using The Observer XT 12 (Noldus, Wageningen, The Netherlands), the cage floor was virtually divided into 3 zones: soiled bedding zone (1/3), center zone with clean bedding (1/3), and clean bedding zone (1/3). The time the pup spent in the soiled bedding zone (1/3) and clean bedding zone (1/3), as well as the number of line crossings, were analyzed. Isolation-induced USV were analyzed using DS (version 3.1.0; [18]). First, the DS object detector using a faster regional convolutional neural network (FASTER-RCNN) was custom-trained using the default CNN Rat Detector YOLO R1 and over 3,000 images of isolation-induced USV obtained through broad-band, high-resolution ultrasound recording of isolation-induced USV emitted during the homing test. Then, all broad-band, high-resolution ultrasound recordings were processed using the custom-trained FASTER-RCNN, isolation-induced USV were detected, and detection files were generated. The accuracy of call detection by DS was verified manually by an experienced user. When necessary, false positives were eliminated and missed isolation-induced USV were included. The total number of isolation-induced USV was calculated for the entire 10-min homing test period. Besides call number, total calling time was assessed.

### Maternal preference test

To assess the preference of *Tph2*^*+/−*^ heterozygous mothers for their *Tph2*^*−/−*^ knockout pups versus *Tph2*^*+/+*^ wildtype littermate controls, a maternal preference test was conducted on P7 under dim red-light conditions (room temperature: 20–21 °C). To this aim, one *Tph2*^*−/−*^ pup and one *Tph2*^*+/+*^ pup from the same litter were placed in separate wired cylinders (diameter: 10.5 cm; height 12 cm) with clean bedding at opposite corners of an open field (60 × 60 × 60 cm). The positions of the *Tph2*^*−/−*^ and *Tph2*^*+/+*^ pups were counter-balanced across tests to avoid positional bias. Immediately after placing the pups, their own mother was placed in the center of the open field, and her behavior was video recorded for 10 min (EQ150, EverFocus, Taipei, Taiwan). Mothers were exposed to all *Tph2*^*−/−*^ versus *Tph2*^*+/+*^ pairs present in their litters, i.e. mothers were repeatedly tested but no pup was used more than once. For behavior scoring using The Observer XT 12 (Noldus, Wageningen, The Netherlands), a 2 cm radius around the wired cylinder was defined. The time the mother spent within this radius around the cylinders with her nose was used to evaluate her preference for *Tph2*^*−/−*^ versus *Tph2*^*+/+*^ pups. To acclimate the *Tph2*^*+/−*^ heterozygous mothers to the experimental setup and procedure, they were individually placed in the open field with two empty cylinders for 10 min the day before the maternal preference test.

### Statistical analysis

To analyze developmental milestones, somatosensory reflexes, thermoregulatory capabilities, and isolation-induced USV, including all acoustic call features, ANOVAs for repeated measurements with the between-subjects factors sex, genotype, and nesting condition, and the within-subject factor development stage, were used. Time spent in the homing test was analyzed using ANOVAs for repeated measurements with the between-subjects factors sex, genotype, and nesting condition, and the within-subject zone. Line crossings and isolation-induced USV emitted during the homing test were compared using ANOVAs with the between-subjects factors sex, genotype, and nesting condition. For the maternal preference test, ANOVAs for repeated measurements with the between-subjects factors sex and nesting condition, and the within-subject preference, were calculated. In case of lack of sphericity, Greenhouse–Geisser correction was applied. ANOVAs were followed by post-hoc analysis when appropriate. A p-value of < 0.050 was considered statistically significant. Statistical analyses were performed using SPSS (version 29, IBM; Armonk, NY, USA). Figures were created using Prism (GraphPad Software, Boston, MA, USA) and BioRender (BioRender, Toronto, Canada).

## Results

### Developmental milestones, somatosensory reflexes, and thermoregulatory capabilities

**Fig. 2:**
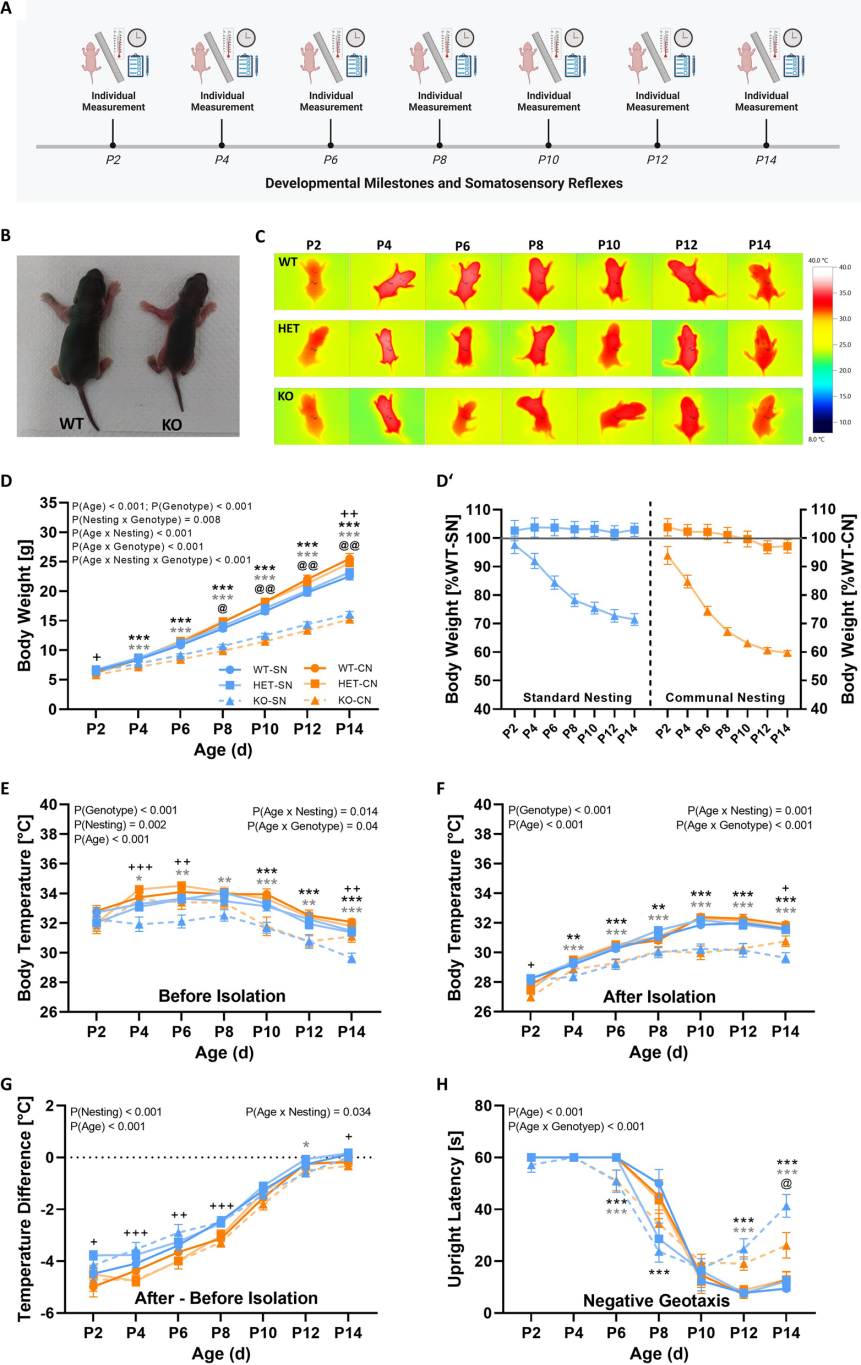
*Tph2* deficiency in rats causes severe growth retardation, together with moderate impairments in somatosensory reflexes and thermoregulatory capabilities, partially aggravated by communal nesting (CN), as compared to standard nesting (SN). (**A**) Overview of the assessment of developmental milestones, somatosensory reflexes, and thermoregulatory capabilities in *Tph2*^*−/−*^ (KO) and *Tph2*^*+/−*^ (HET) rat pups, as compared to *Tph2*^*+/+*^ (WT) littermate controls. (**B**) Exemplary image of WT and KO rat pups on P10. (**C**) Exemplary thermal image of WT, HET, and KO across P2 to P14 obtained during body temperature measurements. (**D**) Absolute body weight of WT, HET, and KO rat pups across P2 to P14, depending on nesting condition. (**D**′) Normalized body weight of HET and KO rat pups across P2 to P14 relative to WT littermate controls, depending on nesting condition. (**E**) Body temperature before the 10-min isolation period for WT, HET, and KO rat pups across P2 to P14, depending on nesting condition. (**F**) Body temperature after the 10-min isolation period for WT, HET, and KO rat pups across P2 to P14, depending on nesting condition. (**G**) Body temperature change during the 10-min isolation period for WT, HET, and KO across P2 to P14, depending on nesting condition. (**H**) Upright latency in the negative geotaxis assay for WT, HET, and KO rat pups across P2 to P14, depending on nesting condition. SN = standard nesting (blue), CN = communal nesting (orange). P = postnatal day. Data are expressed as mean ± SEM. Effect of genotype: ***^(black)^ WT vs. KO p < .001; **^(black)^ WT vs. KO p < .01. ***^(gray)^ HET vs. KO p < .001; *^(gray)^ HET vs. KO p < .01; *^(gray)^ HET vs. KO p < .05. Effect of nesting: +  +  + p < .001; +  + p < .01; + p < .05. Interaction nesting x genotype: @@ p < .01; @ p < .05. N(WT-SN) = 16, N(HET-SN) = 18, N(KO-SN) = 19, N(WT-CN) = 12, N(HET-CN) = 12, N(KO-CN) = 17

*Tph2* deficiency in rats caused severe growth retardation, together with moderate impairments in somatosensory reflexes and thermoregulatory capabilities, partially aggravated by CN, as compared to SN. Developmental milestones and somatosensory reflexes were assessed every second day during the first two weeks of life (Fig. 2A). This included the assessment of body weight gain (Fig. 2B) and thermoregulatory capabilities (Fig. 2C). Throughout the first two weeks of life, body weight gain was strongly affected by genotype (genotype: F_2,82_ = 101.743; p < 0.001; age × genotype: F_2.865,117.462_ = 157.843; p < 0.001). Specifically, body weight increased substantially in *Tph2*^*+/−*^ pups and *Tph2*^*+/+*^ littermate controls, while body weight gain was reduced in *Tph2*^*−/−*^ pups. Whereas there was no genotype difference on P2, differences in body weight started to emerge from P4, resulting in body weight values of *Tph2*^*−/−*^ pups being 20–40% lower by P14 than those observed in *Tph2*^*+/+*^ littermates (Fig. 2D,D′). Surprisingly, the growth retardation phenotype displayed by *Tph2*^*−/−*^ pups was aggravated by CN, as reflected in even further reduced body weight gain in *Tph2*^*−/−*^ pups exposed to CN, as compared to SN (nesting × genotype: F_2,82_ = 5.094; p = 0.008; age × nesting × genotype: F_2.865,117.462_ = 6.563; p < 0.001). Moreover, the aggravated growth retardation phenotype in *Tph2*^*−/−*^ pups exposed to CN is in contrast to the increased body weight gain seen in *Tph2*^*+/−*^ pups and *Tph2*^*+/+*^ littermates exposed to CN, possibly reflecting higher levels of competition between pups under CN, as compared to SN. Of note, a similar pattern was obtained irrespective of whether body weight values were expressed as percentages relative to all *Tph2*^*+/+*^ littermates or separately for *Tph2*^*+/+*^ littermates exposed to SN versus CN, respectively.

Growth retardation in *Tph2*^*−/−*^ pups was associated with impairments in thermoregulatory capabilities. Body temperature was assessed before and after a 10-min social isolation period under room temperature. When measuring body temperature right after removal from the nest before isolation, prominent genotype differences were evident, with *Tph2*^*−/−*^ pups having lower body temperature values than *Tph2*^*+/−*^ pups and *Tph2*^*+/+*^ littermate controls (genotype: F_2,82_ = 21.851; p < 0.001; Fig. 2E). The developmental pattern was similar to body weight gain (age × genotype: F_10.366,424.997_ = 1.906; p = 0.040). While no genotype differences were seen on P2, body temperature differences were evident during later stages of development. However, in contrast to growth retardation, effects of *Tph2* deficiency on thermoregulatory capabilities were not aggravated by CN, as compared to SN (nesting × genotype: F_2,82_ = 0.193; p = 0.825; age × nesting × genotype: F_10.366,424.997_ = 0.894; p = 0.542). A similar result pattern was found after the 10-min isolation period, with prominent genotype differences in body temperature starting at P4 (genotype: F_2,80_ = 45.522; p < 0.001; age × genotype: F_9.315,372.605_ = 4.940; p < 0.001), irrespective of CN (nesting × genotype: F_2,80_ = 0.163; p = 0.850; age × nesting × genotype: F_9.315,372.605_ = 0.794; p = 0.626; Fig. 2F). Comparing the body temperature before and after the 10-min isolation period showed that rat pups typically lost between 3 °C to 6 °C on P2, while the typical temperature loss on P14 was less than 1 °C, reflecting improved thermoregulatory capabilities with age. Improved thermoregulatory capabilities with age were seen irrespective of genotype and CN (genotype: F_2,80_ = 0.695; p = 0.502; age × genotype: F_8.715,348.587_ = 1.150; p = 0.327; nesting × genotype: F_2,80_ = 0.397; p = 0.673; age × nesting × genotype: F_8.715,348.587_ = 0.674; p = 0.727), suggesting that the genotype differences in body temperature evident before isolation were maintained but not exacerbated throughout isolation.

Growth retardation in *Tph2*^*−/−*^ pups was further associated with delays in several developmental milestones (Table 1). Firstly, growth retardation was not only reflected in body weight but also body length (genotype: genotype: F_2,82_ = 35.864; p < 0.001; age × genotype: F_7.685,315.088_ = 73.624; p < 0.001). Moreover, eye opening was delayed in *Tph2*^*−/−*^ pups (genotype: F_2,82_ = 7.905; p = 0.001; age × genotype: F_12,492_ = 6.579; p < 0.001). The visibility of a milk spot in the belly was likewise affected by genotype, with less visible milk spots in *Tph2*^*−/−*^ pups, as compared to *Tph2*^*+/−*^ pups and *Tph2*^*+/+*^ littermate controls, particularly during the first week of life (genotype: F_2,82_ = 2.329; p = 0.104; age × genotype: F_8.171,335.031_ = 4.007; p < 0.001). However, those phenotypes were not modulated by CN (all p-values > 0.50). Pinnae detachment, incisor eruption, and fur development were not affected by genotype (all p-values > 0.50).

**Table 1:** Developmental milestones

Develomental Milestones	Nesting	Genotype	P2	P4	P6	P8	P10	P12	P14
Body Length (cm)	SN	WT	5.26 ± 0.06	5.82 ± 0.07	6.35 ± 0.06	6.85 ± 0.08	7.16 ± 0.06	7.59 ± 0.07	8.02 ± 0.08
###,***,§§§		HET	5.34 ± 0.08	5.88 ± 0.07	6.39 ± 0.06	6.86 ± 0.08	7.21 ± 0.07	7.64 ± 0.08	8.06 ± 0.09
		KO	5.40 ± 0.07	5.85 ± 0.07	6.16 ± 0.06	6.46 ± 0.07	6.63 ± 0.06	6.92 ± 0.10	7.25 ± 0.09
	CN	WT	5.16 ± 0.06	5.82 ± 0.07	6.31 ± 0.06	6.80 ± 0.09	7.27 ± 0.08	7.58 ± 0.09	8.06 ± 0.08
		HET	5.26 ± 0.07	5.83 ± 0.06	6.27 ± 0.04	6.88 ± 0.08	7.29 ± 0.07	7.58 ± 0.07	8.00 ± 0.05
		KO	5.26 ± 0.06	5.64 ± 0.05	5.98 ± 0.04	6.24 ± 0.05	6.49 ± 0.06	6.71 ± 0.05	6.94 ± 0.05
Pinnae Detachment (n)	SN	WT	1.13 ± 0.26	2.00 ± 0.00	1.88 ± 0.09	2.00 ± 0.00	2.00 ± 0.00	2.00 ± 0.00	2.13 ± 0.09
###, + ,$$$		HET	0.61 ± 0.20	1.89 ± 0.08	1.78 ± 0.10	1.94 ± 0.06	2.00 ± 0.00	2.00 ± 0.00	2.28 ± 0.11
		KO	0.84 ± 0.22	1.68 ± 0.11	1.68 ± 0.11	1.79 ± 0.10	2.00 ± 0.00	2.00 ± 0.00	2.05 ± 0.05
	CN	WT	0.25 ± 0.18	2.00 ± 0.00	1.92 ± 0.08	2.00 ± 0.00	2.00 ± 0.00	2.00 ± 0.00	2.00 ± 0.00
		HET	0.17 ± 0.11	1.92 ± 0.08	1.83 ± 0.11	2.00 ± 0.00	2.00 ± 0.00	2.00 ± 0.00	2.08 ± 0.08
		KO	0.06 ± 0.06	1.88 ± 0.08	1.88 ± 0.08	1.94 ± 0.06	2.00 ± 0.00	2.00 ± 0.00	2.00 ± 0.00
Eye Opening (n)	SN	WT	1.00 ± 0.00	1.00 ± 0.00	1.00 ± 0.00	1.00 ± 0.00	1.00 ± 0.00	1.19 ± 0.10	1.88 ± 0.09
###, ***, §§§		HET	1.00 ± 0.00	1.00 ± 0.00	1.00 ± 0.00	1.06 ± 0.06	1.06 ± 0.06	1.11 ± 0.08	1.83 ± 0.09
		KO	1.00 ± 0.00	1.00 ± 0.00	1.00 ± 0.00	1.00 ± 0.00	1.00 ± 0.00	1.00 ± 0.00	1.37 ± 0.11
	CN	WT	1.00 ± 0.00	1.00 ± 0.00	1.00 ± 0.00	1.00 ± 0.00	1.00 ± 0.00	1.00 ± 0.00	1.83 ± 0.11
		HET	1.00 ± 0.00	1.00 ± 0.00	1.00 ± 0.00	1.00 ± 0.00	1.00 ± 0.00	1.00 ± 0.00	1.92 ± 0.08
		KO	1.00 ± 0.00	1.00 ± 0.00	1.00 ± 0.00	1.00 ± 0.00	1.00 ± 0.00	1.00 ± 0.00	1.59 ± 0.12
Incisor Eruption (n)	SN	WT	0.00 ± 0.00	0.00 ± 0.00	0.00 ± 0.00	0.19 ± 0.10	1.56 ± 0.20	2.00 ± 0.00	2.00 ± 0.00
###		HET	0.00 ± 0.00	0.00 ± 0.00	0.00 ± 0.00	0.22 ± 0.13	1.39 ± 0.20	2.00 ± 0.00	2.00 ± 0.00
		KO	0.00 ± 0.00	0.00 ± 0.00	0.00 ± 0.00	0.32 ± 0.15	1.63 ± 0.16	2.00 ± 0.00	2.00 ± 0.00
	CN	WT	0.00 ± 0.00	0.00 ± 0.00	0.00 ± 0.00	0.17 ± 0.11	2.00 ± 0.00	2.00 ± 0.00	2.00 ± 0.00
		HET	0.00 ± 0.00	0.00 ± 0.00	0.00 ± 0.00	0.00 ± 0.00	1.50 ± 0.23	2.00 ± 0.00	2.00 ± 0.00
		KO	0.00 ± 0.00	0.00 ± 0.00	0.00 ± 0.00	0.00 ± 0.00	1.65 ± 0.19	2.00 ± 0.00	2.00 ± 0.00
Fur Development (n)	SN	WT	0.00 ± 0.00	0.34 ± 0.06	0.72 ± 0.10	1.47 ± 0.10	2.25 ± 0.11	2.81 ± 0.06	3.00 ± 0.00
###, $$$		HET	0.00 ± 0.00	0.39 ± 0.05	0.69 ± 0.09	1.56 ± 0.04	2.39 ± 0.09	2.94 ± 0.04	3.00 ± 0.00
		KO	0.00 ± 0.00	0.45 ± 0.04	0.74 ± 0.10	1.55 ± 0.08	2.34 ± 0.08	2.76 ± 0.06	2.97 ± 0.03
	CN	WT	0.04 ± 0.04	0.42 ± 0.06	0.92 ± 0.14	1.54 ± 0.04	2.50 ± 0.00	2.83 ± 0.07	3.00 ± 0.00
		HET	0.00 ± 0.00	0.46 ± 0.04	1.00 ± 0.14	1.67 ± 0.07	2.55 ± 0.05	2.88 ± 0.07	3.00 ± 0.00
		KO	0.00 ± 0.00	0.38 ± 0.05	0.88 ± 0.12	1.59 ± 0.07	2.35 ± 0.09	2.68 ± 0.06	2.97 ± 0.03
Milk Spot (n)	SN	WT	3.00 ± 0.00	2.81 ± 0.14	2.94 ± 0.06	2.50 ± 0.13	2.00 ± 0.20	0.69 ± 0.18	0.06 ± 0.06
###, §§§, $		HET	2.89 ± 0.11	2.94 ± 0.06	3.00 ± 0.00	2.72 ± 0.11	2.11 ± 0.16	0.67 ± 0.16	0.00 ± 0.00
		KO	2.89 ± 0.11	2.79 ± 0.14	2.89 ± 0.07	2.58 ± 0.14	2.16 ± 0.19	1.21 ± 0.25	0.26 ± 0.13
	CN	WT	3.00 ± 0.00	2.92 ± 0.08	2.83 ± 0.11	2.00 ± 0.37	1.25 ± 0.37	0.67 ± 0.14	0.00 ± 0.00
		HET	3.00 ± 0.00	3.00 ± 0.00	3.00 ± 0.00	2.42 ± 0.34	1.58 ± 0.38	0.75 ± 0.18	0.00 ± 0.00
		KO	2.94 ± 0.06	2.53 ± 0.21	2.76 ± 0.18	2.12 ± 0.24	2.35 ± 0.17	1.47 ± 0.26	0.24 ± 0.11

Data are expressed as mean ± SEM. P = postnatal day. (n) = Semi-quantitative rating (0 = Absent, 1 = Uncertain, 2 = Present and 3 = Evident). Effect of age: ###p < .001. Effect of genotype: ***p < .001. Effect of nesting: + p < .05. Interaction age x genotype: §§§p < .001. Interaction age x nesting: $$$p < .001; $p < .05

Finally, the developmental delay displayed by *Tph2*^*−/−*^ pups was associated with a broad variety of alterations in somatosensory reflexes (Table 2). Firstly, the grasping reflex was found to be more prominent in *Tph2*^*−/−*^ pups than in *Tph2*^*+/−*^ pups and *Tph2*^*+/+*^ littermate controls (genotype: F_2,82_ = 8.046; p = 0.001; age × genotype: F_6.982,286.243_ = 3.902; p < 0.001). Secondly, forelimb placing was reduced in *Tph2*^*−/−*^ pups (genotype: F_2,82_ = 131.532; p < 0.001; age × genotype: F_10.137,415.624_ = 4.782; p < 0.001; Supplementary Fig. 1A). Thirdly, the righting reflex was affected by genotype, with *Tph2*^*−/−*^ pups being able to flip over onto the abdomen faster, particularly during the first week of life, possibly because of their lower body weight (genotype: F_2,82_ = 3.082; p = 0.051; age × genotype: F_6.286,257.712_ = 2.343; p = 0.030; Supplementary Fig. 1B). Fourthly, level screen was slightly impaired in *Tph2*^*−/−*^ pups during the first week of life (genotype: F_2,82_ = 0.160; p = 0.852; age × genotype: F_8.697,356.580_ = 3.765; p < 0.001; Supplementary Fig. 1C). Fifthly, negative geotaxis was impaired in *Tph2*^*−/−*^ pups. While all genotypes started to turn around on a 45° grid to reach an upright position in the second week of life, there was evidence for a regression in *Tph2*^*−/−*^ pups on P12 and P14 (genotype: F_2,82_ = 2.109; p = 0.128; age × genotype: F_7.053,289.161_ = 11.043; p < 0.001; Fig. 2H). Sixthly, an increase in vertical screen behavior was observed in *Tph2*^*−/−*^ pups (genotype: F_2,82_ = 7.281; p = 0.001; age × genotype: F_7.968,326.676_ = 1.798; p = 0.077; Supplementary Fig. 1D). Seventhly, *Tph2*^*−/−*^ pups needed more time to withdraw from an elevated edge in the cliff avoidance task during the second week of life (genotype: F_2,82_ = 0.681; p = 0.509; age × genotype: F_10.595,434.396_ = 2.118; p = 0.020). Finally, *Tph2*^*−/−*^ pups were less able to hold onto a bar resulting in a reduced bar holding time, especially during the second week of life (genotype: F_2,82_ = 6.745; p = 0.002; age × genotype: F_7.282,298.548_ = 2.676; p = 0.010). However, except for forelimb placing, alterations in somatosensory reflexes were not modulated by CN (nesting × genotype: F_2,82_ = 4.830; p = 0.010; all other p-values > 0.50). Sex had no prominent modulatory effects on the alterations caused by *Tph2* deficiency in developmental milestones, somatosensory reflexes, or thermoregulatory capabilities, except for grasping reflex and forelimb placing (not shown in detail).

**Table 2:** Somatosensory reflexes

Somatosensory Reflexes	Nesting	Genotype	P2	P4	P6	P8	P10	P12	P14
Grasping Reflex (n)	SN	WT	2.19 ± 0.31	2.13 ± 0.24	2.38 ± 0.22	2.56 ± 0.16	2.94 ± 0.06	3.00 ± 0.00	3.00 ± 0.00
###, **, §§§, €€		HET	2.67 ± 0.18	2.28 ± 0.16	2.06 ± 0.25	2.67 ± 0.16	2.94 ± 0.06	3.00 ± 0.00	3.00 ± 0.00
		KO	2.74 ± 0.17	2.89 ± 0.11	2.68 ± 0.15	2.89 ± 0.07	3.00 ± 0.00	2.95 ± 0.05	2.95 ± 0.05
	CN	WT	2.75 ± 0.18	1.92 ± 0.34	2.50 ± 0.19	2.75 ± 0.13	2.92 ± 0.08	3.00 ± 0.00	3.00 ± 0.00
		HET	2.42 ± 0.23	2.33 ± 0.28	2.33 ± 0.22	2.67 ± 0.19	3.00 ± 0.00	3.00 ± 0.00	3.00 ± 0.00
		KO	2.71 ± 0.19	2.94 ± 0.06	2.94 ± 0.06	3.00 ± 0.00	2.94 ± 0.06	3.00 ± 0.00	3.00 ± 0.00
Forelimb Placing (n)	SN	WT	0.31 ± 0.15	1.06 ± 0.30	2.06 ± 0.19	2.44 ± 0.22	2.75 ± 0.11	2.88 ± 0.09	2.81 ± 0.10
###,***,%%,§§§,$$,@,€€		HET	0.61 ± 0.18	0.94 ± 0.25	2.28 ± 0.21	2.56 ± 0.18	2.94 ± 0.06	3.00 ± 0.00	2.94 ± 0.06
		KO	0.05 ± 0.05	0.21 ± 0.10	0.79 ± 0.24	1.26 ± 0.18	1.84 ± 0.19	2.05 ± 0.25	1.74 ± 0.21
	CN	WT	0.25 ± 0.13	1.92 ± 0.36	2.67 ± 0.14	2.67 ± 0.26	2.83 ± 0.17	3.00 ± 0.00	3.00 ± 0.00
		HET	0.58 ± 0.23	2.08 ± 0.29	2.58 ± 0.19	3.00 ± 0.00	2.75 ± 0.18	3.00 ± 0.00	3.00 ± 0.00
		KO	0.00 ± 0.00	0.06 ± 0.06	0.76 ± 0.25	0.59 ± 0.19	1.29 ± 0.31	1.94 ± 0.23	2.24 ± 0.18
Righting Reflex (s)	SN	WT	24.34 ± 4.37	21.77 ± 6.36	3.61 ± 0.86	1.17 ± 0.11	2.22 ± 0.84	0.83 ± 0.08	0.74 ± 0.09
###,§,Ω		HET	25.63 ± 4.52	10.97 ± 2.53	4.86 ± 3.25	2.21 ± 1.06	0.89 ± 0.07	0.86 ± 0.08	0.65 ± 0.06
		KO	11.74 ± 3.18	4.87 ± 1.80	1.68 ± 0.25	1.40 ± 0.11	1.98 ± 0.44	1.78 ± 0.58	1.16 ± 0.16
	CN	WT	19.31 ± 4.47	23.31 ± 7.32	2.02 ± 0.26	7.56 ± 4.93	4.59 ± 3.51	5.28 ± 4.62	0.68 ± 0.10
		HET	17.18 ± 3.69	24.8 ± 6.28	1.56 ± 0.27	1.21 ± 0.22	3.15 ± 1.79	0.82 ± 0.08	0.57 ± 0.08
		KO	21.34 ± 4.25	11.29 ± 4.47	1.66 ± 0.15	5.20 ± 3.43	5.03 ± 3.44	1.42 ± 0.29	0.93 ± 0.17
Level Screen (n)	SN	WT	0.31 ± 0.12	1.31 ± 0.15	2.13 ± 0.13	2.25 ± 0.14	2.75 ± 0.11	3.00 ± 0.00	3.00 ± 0.00
###,§§§,$		HET	0.28 ± 0.14	1.28 ± 0.16	1.83 ± 0.15	2.44 ± 0.15	2.67 ± 0.11	2.94 ± 0.06	3.00 ± 0.00
		KO	0.42 ± 0.12	0.74 ± 0.20	1.89 ± 0.15	2.63 ± 0.11	2.79 ± 0.10	3.00 ± 0.00	3.00 ± 0.00
	CN	WT	0.42 ± 0.15	1.17 ± 0.17	1.83 ± 0.11	2.00 ± 0.12	2.75 ± 0.13	2.92 ± 0.08	3.00 ± 0.00
		HET	0.58 ± 0.15	1.25 ± 0.13	1.67 ± 0.14	2.42 ± 0.15	2.42 ± 0.15	2.92 ± 0.08	3.00 ± 0.00
		KO	0.53 ± 0.15	0.82 ± 0.15	1.41 ± 0.19	2.41 ± 0.12	2.65 ± 0.12	3.00 ± 0.00	3.00 ± 0.00
Negative Geotaxis (s)	SN	WT	60.00 ± 0.00	60.00 ± 0.00	60.00 ± 0.00	49.94 ± 5.41	12.4 ± 3.82	7.79 ± 0.80	9.43 ± 0.93
###,§§§		HET	60.00 ± 0.00	60.00 ± 0.00	60.00 ± 0.00	28.62 ± 6.11	16.2 ± 4.78	7.69 ± 0.85	12.26 ± 1.88
		KO	57.15 ± 2.85	60.00 ± 0.00	50.84 ± 4.26	23.75 ± 4.14	17.09 ± 1.89	24.73 ± 3.90	41.33 ± 4.34
	CN	WT	60.00 ± 0.00	60.00 ± 0.00	60.00 ± 0.00	44.96 ± 6.51	14.61 ± 4.70	7.16 ± 1.55	12.89 ± 3.12
		HET	60.00 ± 0.00	60.00 ± 0.00	60.00 ± 0.00	43.56 ± 7.13	12.18 ± 4.63	8.84 ± 1.72	12.89 ± 2.94
		KO	60.00 ± 0.00	60.00 ± 0.00	51.18 ± 4.00	34.5 ± 5.29	19.47 ± 3.26	19.06 ± 2.56	26.12 ± 4.91
Vertical Screen (s)	SN	WT	1.03 ± 0.40	1.74 ± 0.32	8.79 ± 1.97	14.41 ± 2.77	18.51 ± 4.07	35.58 ± 5.59	49.23 ± 4.99
###,**		HET	0.79 ± 0.25	2.59 ± 0.40	12.71 ± 3.43	14.39 ± 3.13	20.38 ± 3.74	30.54 ± 4.46	51.47 ± 4.44
		KO	1.94 ± 0.55	6.32 ± 1.66	15.07 ± 3.59	12.58 ± 2.46	27.23 ± 5.80	38.21 ± 5.13	58.07 ± 1.72
	CN	WT	0.86 ± 0.37	2.63 ± 0.65	11.15 ± 1.92	11.82 ± 2.14	24.04 ± 5.92	25.1 ± 6.35	60.00 ± 0.00
		HET	0.00 ± 0.00	5.73 ± 2.35	9.88 ± 1.89	21.97 ± 4.12	11.70 ± 2.56	23.9 ± 5.48	56.51 ± 3.49
		KO	0.32 ± 0.15	4.15 ± 1.24	11.06 ± 2.73	24.56 ± 5.47	30.94 ± 5.79	45.71 ± 5.03	60.00 ± 0.00
Cliff Avoidance (s)	SN	WT	25.61 ± 2.39	30.00 ± 0.00	28.22 ± 1.66	15.88 ± 2.78	9.65 ± 2.10	11.18 ± 2.23	7.92 ± 2.35
###,§		HET	27.47 ± 1.75	28.88 ± 1.12	27.09 ± 1.62	20.3 ± 2.56	13.06 ± 2.15	9.00 ± 1.73	10.05 ± 2.63
		KO	28.49 ± 1.51	21.63 ± 2.91	22.36 ± 2.72	20.59 ± 2.64	11.24 ± 2.55	16.22 ± 3.81	12.11 ± 2.66
	CN	WT	21.43 ± 3.66	30.00 ± 0.00	27.82 ± 2.18	17.29 ± 3.37	10.59 ± 2.34	11.52 ± 1.48	8.25 ± 2.81
		HET	27.62 ± 2.36	30.00 ± 0.00	28.09 ± 1.69	25.13 ± 2.16	13.43 ± 3.06	8.91 ± 1.92	7.10 ± 1.94
		KO	23.45 ± 2.61	28.84 ± 1.16	25.33 ± 2.52	20.16 ± 3.01	15.36 ± 2.92	12.96 ± 3.04	7.20 ± 1.78
Bar Holding (s)	SN	WT	2.87 ± 0.45	12.91 ± 4.67	35.48 ± 4.61	32.62 ± 4.55	14.68 ± 2.57	11.64 ± 1.59	14.75 ± 3.07
###,**,§		HET	2.86 ± 0.32	11.99 ± 2.78	32.15 ± 4.97	41.73 ± 4.62	19.73 ± 2.97	10.54 ± 0.87	15.62 ± 2.46
		KO	3.31 ± 0.72	11.58 ± 2.95	25.81 ± 5.61	19.45 ± 2.77	8.94 ± 1.26	7.14 ± 1.45	7.09 ± 1.36
	CN	WT	1.58 ± 0.32	5.09 ± 1.02	37.67 ± 5.88	29.26 ± 5.00	16.01 ± 3.18	10.67 ± 1.51	10.45 ± 1.63
		HET	1.88 ± 0.26	8.10 ± 2.06	28.26 ± 5.73	29.17 ± 3.89	14.29 ± 1.99	9.01 ± 1.16	13.72 ± 2.88
		KO	5.73 ± 3.41	12.88 ± 3.78	21.30 ± 4.99	22.66 ± 4.48	15.23 ± 2.73	5.07 ± 1.13	6.11 ± 1.14
Auditory Startle (s)	SN	WT	0.00 ± 0.00	0.06 ± 0.06	0.00 ± 0.00	0.00 ± 0.00	0.00 ± 0.00	0.63 ± 0.29	2.31 ± 0.30
###, + ,Ω		HET	0.06 ± 0.06	0.11 ± 0.11	0.11 ± 0.11	0.06 ± 0.06	0.00 ± 0.00	0.28 ± 0.19	2.17 ± 0.25
		KO	0.00 ± 0.00	0.05 ± 0.05	0.05 ± 0.05	0.05 ± 0.05	0.00 ± 0.00	0.26 ± 0.18	2.47 ± 0.22
	CN	WT	0.00 ± 0.00	0.00 ± 0.00	0.00 ± 0.00	0.00 ± 0.00	0.00 ± 0.00	0.00 ± 0.00	2.00 ± 0.35
		HET	0.00 ± 0.00	0.00 ± 0.00	0.00 ± 0.00	0.00 ± 0.00	0.00 ± 0.00	0.00 ± 0.00	2.17 ± 0.34
		KO	0.00 ± 0.00	0.00 ± 0.00	0.00 ± 0.00	0.00 ± 0.00	0.00 ± 0.00	0.06 ± 0.06	2.18 ± 0.32

Data are expressed as mean ± SEM. P = postnatal day. (n) = Semi-quantitative rating (0 = Absent, 1 = Uncertain, 2 = Present and 3 = Evident). Effect of age: ###p < .001. Effect of genotype: ***p < .001; **p < .01. Effect of nesting: + p < .05. Effect of sex: %%p < .01. Interaction age x genotype: §§§p < .001; §p < .05. Interaction age x nesting: $$p < .01; $p < .05. Interaction nesting x genotype: @p < .05. Interaction sex x genotype: €€p < .01. Interaction age x nesting x sex: Ω p < .05.

Taken together, *Tph2* deficiency led to severe growth retardation, as reflected in reduced body weight gain. Moreover, *Tph2* deficiency was associated with impairments in thermoregulatory capabilities and a delay in developmental milestones, most notably eye opening. While CN aggravated the growth retardation phenotype, it did not aggravate the impairments in thermoregulatory capabilities nor the delay in developmental milestones associated with *Tph2* deficiency. The effects of *Tph2* deficiency on somatosensory reflexes were moderate and mostly not modulated by CN.

### Isolation-induced pup ultrasonic vocalizations

**Fig. 3:**
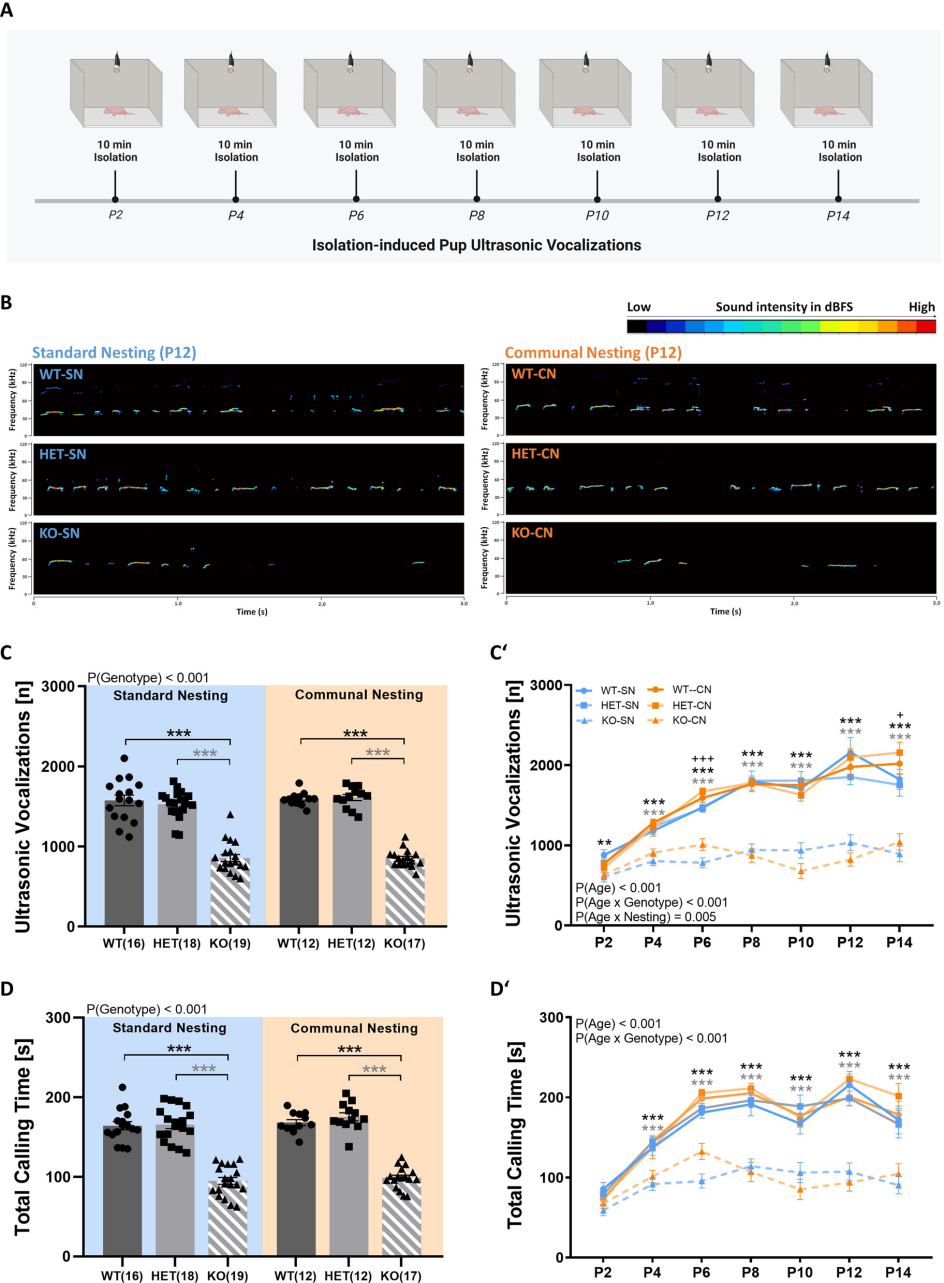
*Tph2* deficiency in rats leads to severe deficits in socio-affective communication during the first two weeks of life, as evidenced by reduced call emission rates. (**A**) Overview of the assessment of isolation-induced ultrasonic vocalizations in *Tph2*^*−/−*^ (KO) and *Tph2*^*+/−*^ (HET) rat pups, as compared to *Tph2*^*+/+*^ (WT) littermate controls. (**B**) Exemplary spectrograms of isolation-induced ultrasonic vocalizations emitted by WT, HET, and KO on P12, depending on nesting condition. (**C**) Average number of isolation-induced ultrasonic vocalizations emitted across P2 to P14. (**D**) Average total calling time across P2 to P14. Developmental trajectories of (**C**′) the number of isolation-induced ultrasonic vocalizations and (**D**′) total calling time across P2 to P14. SN = standard nesting (blue), CN = communal nesting (orange). P = postnatal day. Data are expressed as mean ± SEM. Effect of genotype: ***^(black)^ WT vs. KO p < .001; **^(black)^ WT vs. KO p < .01. ***^(gray)^ HET vs. KO p < .001; Effect of nesting: + p < .05. N(WT-SN) = 16, N(HET-SN) = 18, N(KO-SN) = 19, N(WT-CN) = 12, N(HET-CN) = 12, N(KO-CN) = 17

*Tph2* deficiency in rats led to severe deficits in socio-affective communication during the first two weeks of life, as evidenced by reduced call emission rates, regardless of CN. Socio-affective communication in pups was assessed through isolation-induced USV during the 10-min isolation period every second day (Fig. 3A; for exemplary spectrograms, please see Fig. 3B). The average number of calls emitted over the 7 test days was reduced in *Tph2*^*−/−*^ pups, as compared *Tph2*^*+/−*^ pups and *Tph2*^*+/+*^ littermate controls. This reduction was seen irrespective of CN (genotype: F_2,82_ = 198.971; p < 0.001; nesting × genotype: F_2,82_ = 0.334; p = 0.717; Fig. 3C). While *Tph2*^*+/−*^ pups and *Tph2*^*+/+*^ littermates emitted ~ 1500–1600 isolation-induced USV on average under SN conditions, *Tph2*^*−/−*^ pups emitted only ~ 800–900 isolation-induced USV. This strong reduction of ~ 50% in *Tph2*^*−/−*^ pups was also seen under CN conditions. Whereas isolation-induced USV emission rates again ranged between ~ 1500–1600 in *Tph2*^*+/−*^ pups and *Tph2*^*+/+*^ littermates, CN did not ameliorate the socio-affective communication deficit displayed by *Tph2*^*−/−*^ pups and their emission rate remained at ~ 800–900 isolation-induced USV.

When analyzing the developmental trajectories, it was evident that this phenotype emerged around P2. While isolation-induced USV emission rates increased substantially in *Tph2*^*+/−*^ pups and *Tph2*^*+/+*^ littermate controls across development from ~ 700–900 USV on P2 to ~ 1700–2200 USV on P14, there was no such prominent increase seen in *Tph2*^*−/−*^ pups. Isolation-induced USV emission increased only slightly from ~ 600–700 USV on P2 to ~ 800–1100 USV on P14. This difference in the developmental trajectories resulted in very prominent genotype differences in isolation-induced USV emission rates in the second week of life (age × genotype: F_8.778,359.913_ = 10.869; p < 0.001). CN did not affect the strong effect of *Tph2* deficiency on developmental trajectories (age × nesting × genotype: F_8.778,359.913_ = 1.112; p = 0.354; Fig. 3C′). In correspondence with the number of calls, total calling time showed a similar pattern. Across the 7 test days, *Tph2*^*−/−*^ pups spent less time calling than *Tph2*^*+/−*^ pups and *Tph2*^*+/+*^ littermates (genotype: F_2,82_ = 170.602; p < 0.001; nesting × genotype: F_2,82_ = 0.113; p = 0.893; Fig. 3D). Starting from P4, total calling time was lower in *Tph2*^*−/−*^ pups, as compared to *Tph2*^*+/−*^ pups and *Tph2*^*+/+*^ littermates. Similar to call numbers, most prominent genotype differences were seen during the second week of life (age × genotype: F_9.019,359.797_ = 6.318; p < 0.001; age × nesting × genotype: F_9.019,359.797_ = 1.020; p = 0.424; Fig. 3D′). Of note, CN did not affect isolation-induced USV emission rates and total calling time (all p-values > 0.50). Sex had a mild modulatory effect on the alterations caused by *Tph2* deficiency in isolation-induced USV emission rates and total calling time, with stronger reductions in male than female *Tph2*^*−/−*^ pups (not shown in detail).

**Fig. 4:**
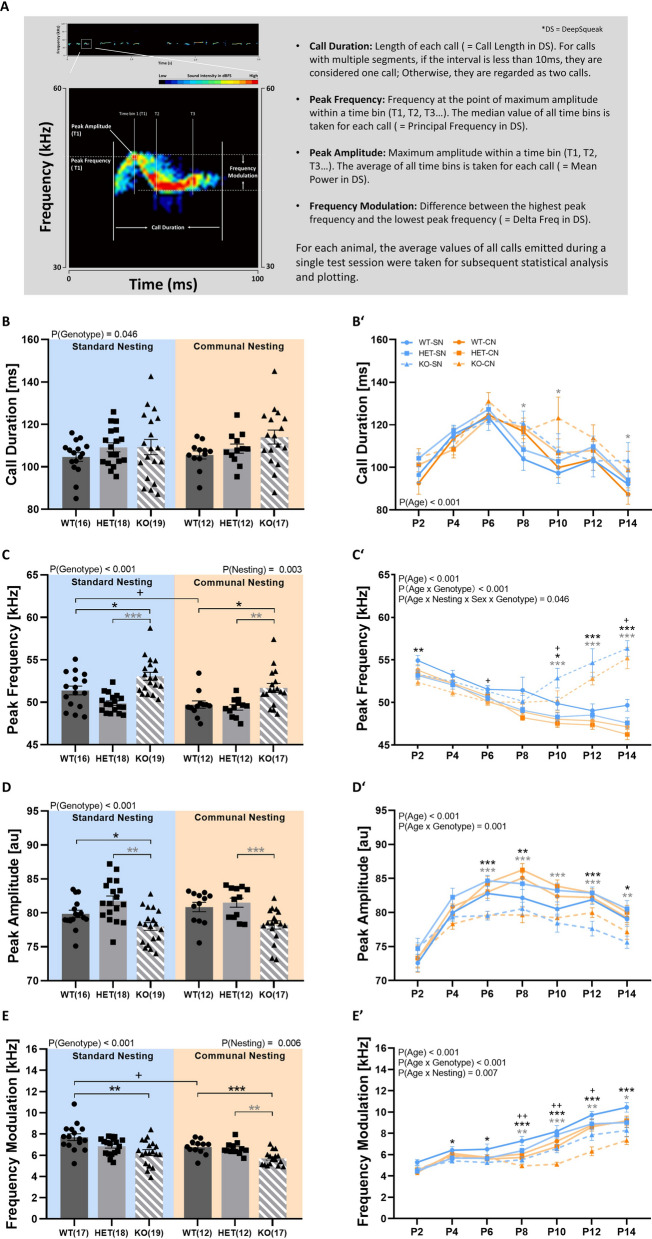
*Tph2* deficiency in rats is results in changes in the acoustic features of isolation-induced ultrasonic vocalizations. (**A**) Overview of the acoustic features of isolation-induced ultrasonic vocalizations assessed in *Tph2*^*−/−*^ (KO) and *Tph2*^*+/−*^ (HET) rat pups, as compared to *Tph2*^*+/+*^ (WT) littermate controls: call duration, peak frequency, peak amplitude, and frequency modulation. (**B**) Average call duration across P2 to P14. (**C**) Average peak frequency across P2 to P14. (**D**) Average peak amplitude across P2 to P14. (**E**) Average frequency modulation across P2 to P14. Developmental trajectories of (**B**′) call duration, (**C**′) peak frequency, (**D**′) peak amplitude, and (**E**′) frequency modulation of isolation-induced ultrasonic vocalizations emitted across P2 to P14. SN = standard nesting (blue), CN = communal nesting (orange). P = postnatal day. Data are expressed as means ± SEM. Effect of genotype: ***^(black)^ WT vs. KO p < .001; **^(black)^ WT vs. KO p < .01; *^(black)^ WT vs. KO p < .05. ***^(gray)^ HET vs. KO p < .001; **^(gray)^ HET vs. KO p < .01; *^(gray)^ HET vs. KO p < .05. Effect of nesting: +  +  + p < .001; + p < .05. N(WT-SN) = 16, N(HET-SN) = 18, N(KO-SN) = 19, N(WT-CN) = 12, N(HET-CN) = 12, N(KO-CN) = 17

The deficits in socio-affective communication driven by *Tph2* deficiency in rats were also reflected in alterations of acoustic features of USV emitted by *Tph2*^*−/−*^ pups. This included all four main acoustic features, i.e. call duration, peak frequency, peak amplitude, and frequency modulation (Fig. 4A). *Tph2*^*−/−*^ pups emitted slightly longer isolation-induced USV than *Tph2*^*+/−*^ pups and *Tph2*^*+/+*^ littermate controls, irrespective of CN (genotype: F_2,82_ = 3.209; p = 0.046; age × genotype: F_9.157,375.450_ = 0.943; p = 0.489; nesting × genotype: F_2,82_ = 0.326; p = 0.722; age × nesting × genotype: F_9.157,375.450_ = 0.763; p = 0.653; Fig. 4B, B′). Moreover, isolation-induced USV emitted by *Tph2*^*−/−*^ pups were characterized by higher peak frequency under SN and CN conditions (genotype: F_2,82_ = 18.358; p < 0.001; nesting × genotype: F_2,82_ = 0.895; p = 0.413; Fig. 4C). This genotype difference in peak frequency was driven by a prominent alteration in the developmental trajectories. While there was a continuous gradual decrease in peak frequency seen across development in *Tph2*^*+/−*^ pups and *Tph2*^*+/+*^ littermates, a U-shaped developmental pattern was evident in *Tph2*^*−/−*^ pups. In the latter, isolation-induced USV with relatively low peak frequencies were seen during the first week of life similar to *Tph2*^*+/−*^ pups and *Tph2*^*+/+*^ littermates, yet a rather abrupt increase in peak frequency occurred around P10 in *Tph2*^*−/−*^ pups. Due to a further increase in *Tph2*^*−/−*^ pups on P12 and P14, genotype differences in peak frequencies reached up to 8 kHz on P14 (age × genotype: F_8.847,362.724_ = 18.412; p < 0.001; age × nesting × genotype: F_8.847,362.724_ = 0.477; p = 0.887; Fig. 4C′). In contrast to peak frequency, *Tph2*^*−/−*^ pups emitted isolation-induced USV characterized by lower peak amplitudes, as compared to *Tph2*^*+/−*^ pups and *Tph2*^*+/+*^ littermates (genotype: F_2,82_ = 15.716; p < 0.001; nesting × genotype: F_2,82_ = 0.557; p = 0.575; Fig. 4D). Genotype differences started to emerge from around P6 and prominent differences were evident throughout the second week of life (age × genotype: F_8.565,351.175_ = 3.137; p = 0.001; age × nesting × genotype: F_8.565,351.175_ = 0.766; p = 0.642; Fig. 4D′). Finally, frequency modulation of isolation-induced USV emitted by *Tph2*^*−/−*^ pups was reduced, regardless of CN (genotype: F_2,82_ = 14.497; p < 0.001; nesting × genotype: F_2,82_ = 1.179; p = 0.313; Fig. 4E). The phenotype started to emerge around P4 and persisted until P14 (age × genotype: F_6.394,262.145_ = 4.389; p < 0.001; age × nesting × genotype: F_6.394,262.145_ = 1.158; p = 0.329; Fig. 4E′). Of note, CN led to genotype-independent alterations in the acoustic features of isolation-induced USV. Specifically, reductions in peak frequency (nesting: F_1,82_ = 9.356; p = 0.003; age × nesting: F_4.423,362.724_ = 0.757; p = 0.566; Fig. 4C′) and frequency modulation (nesting: F_1,82_ = 8.002; p = 0.006; age × nesting: F_3.197,262.145_ = 3.958; p = 0.007; Fig. 4E′) were seen in the pups exposed to CN, particularly in the later phase of development (all other p-values > 0.50). Sex had a mild modulatory effect on the alterations caused by *Tph2* deficiency in peak frequency, with more prominent genotype differences in male than female *Tph2*^*−/−*^ pups under CN but not SN conditions (not shown in detail).

**Fig. 5:**
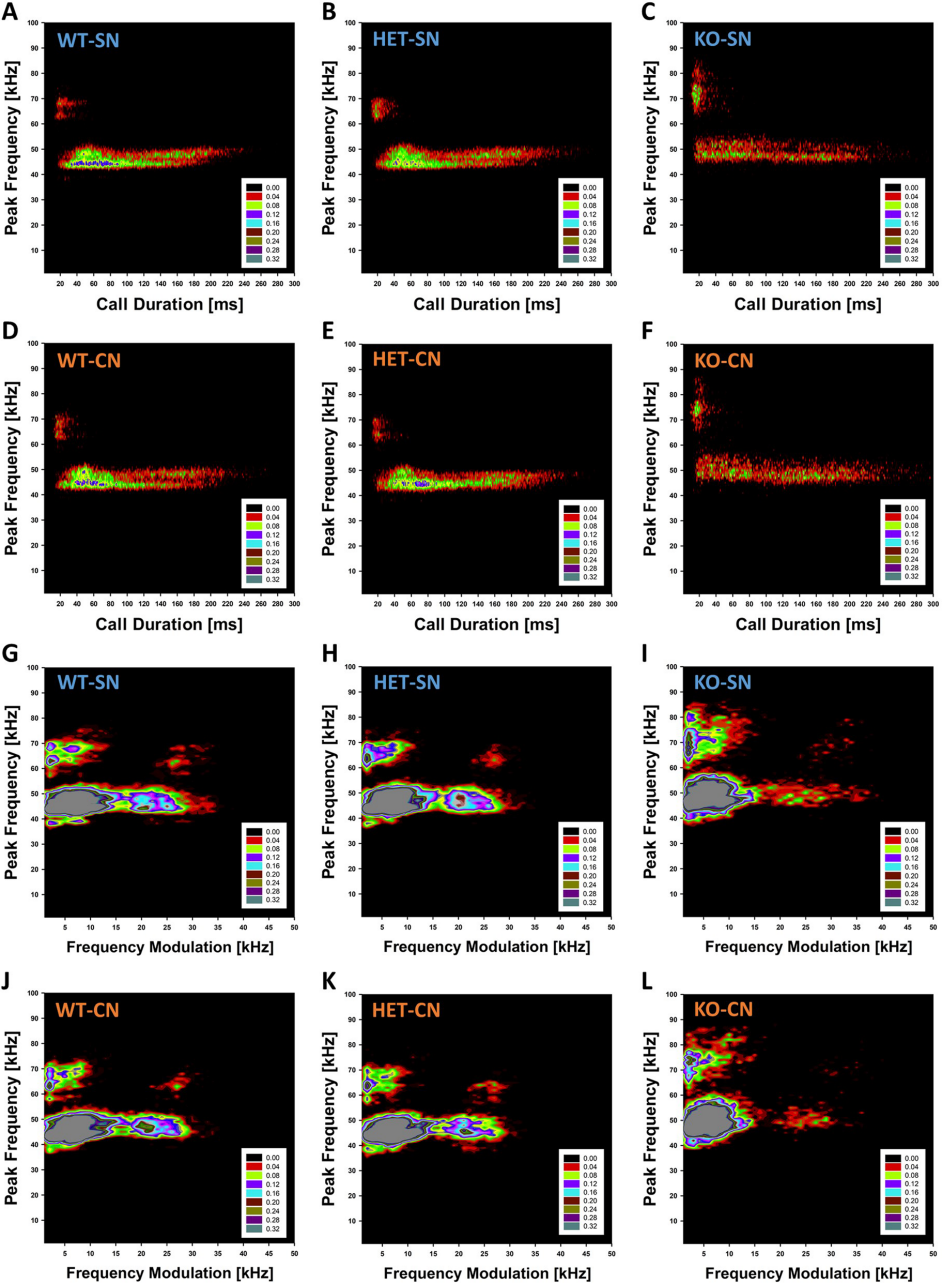
*Tph2* deficiency in rats is associated with changes in the clustering of subtypes of isolation-induced ultrasonic vocalizations. (**A-L**) Density plots depicting the distribution of individual isolation-induced ultrasonic vocalizations in *Tph2*^*−/−*^ (KO) and *Tph2*^*+/−*^ (HET) rat pups, as compared to *Tph2*^*+/+*^ (WT) littermate controls, depending on nesting condition, i.e. WT-SN (**A**, **G**; ~ 35,000 calls), WT-CN (**D**, **J**; ~ 24,000 calls), HET-SN (**B**, **H**; ~ 34,000 calls), HET-CN (**E**, **K**; ~ 25,000 calls), KO-SN (**C**, **I**; ~ 20,000 calls), and KO-CN (**F**, **L**; ~ 14,000 calls). SN = standard nesting (blue), CN = communal nesting (orange). Color coding reflects frequencies as percentages. Individual isolation-induced ultrasonic vocalizations depicted here were recorded on postnatal day 12

Moreover, *Tph2* deficiency in rats affected call clustering. Detailed spectrographic analyses of more than 150,000 individual isolation-induced USV on P12 revealed multiple clusters of call subtypes. In *Tph2*^*+/+*^ littermate controls, two call clusters were revealed by plotting peak frequency against call duration. One prominent cluster was characterized by relatively low peak frequencies roughly between 40 and 50 kHz. A second cluster was characterized by relatively high peak frequencies roughly between 60 and 70 kHz. Call durations in the low-frequency cluster were much longer than in the high-frequency cluster. Although both clusters were present in *Tph2*^*−/−*^ pups, the typical peak frequency associated with each of the two clusters was shifted up by ~ 5 kHz. Moreover, the relative prevalence of the two call clusters was affected by genotype. While the low-frequency cluster was much more prominent than the high-frequency cluster in *Tph2*^*+/+*^ littermates, this difference in relative prevalence was smaller in *Tph2*^*−/−*^ pups due to a more prominent high-frequency cluster, ranging roughly from 65 to 85 kHz. As in *Tph2*^*+/+*^ littermates, call durations in the low-frequency cluster were much longer than in the high-frequency cluster. Call clustering in *Tph2*^*+/−*^ pups was more similar to *Tph2*^*+/+*^ littermates than *Tph2*^*−/−*^ pups. CN slightly reduced the prevalence of the high-frequency cluster (Fig. 5A-F).

When plotting peak frequency against frequency modulation, four call clusters were revealed in *Tph2*^*+/+*^ littermate controls. For the low-frequency cluster, two call subtypes were present, differing in frequency modulation. Frequency modulation was either below ~ 15 kHz or above ~ 20 kHz, ranging up to ~ 35 kHz. For the high-frequency cluster, another two call subtypes were present, again differing in frequency modulation. Frequency modulation was either below ~ 15 kHz or above ~ 25 kHz, ranging up to ~ 35 kHz. The low-frequency cluster characterized by low levels of frequency modulation was most prevalent. The high-frequency cluster characterized by high levels of frequency modulation was least prevalent. While all four call clusters were present in *Tph2*^*−/−*^ pups, the two call clusters characterized by high levels of frequency modulation were less prevalent than in *Tph2*^*+/+*^ littermates. In particular, the high-frequency cluster characterized by high levels of frequency modulation was almost absent. This indicates that the increased prevalence of the high-frequency cluster in *Tph2*^*−/−*^ pups compared to *Tph2*^*+/+*^ littermates was exclusively driven by isolation-induced USV characterized by low levels of frequency modulation, i.e. below 15 kHz. Again, call clustering in *Tph2*^*+/−*^ pups was more similar to *Tph2*^*+/+*^ littermates than *Tph2*^*−/−*^ pups. CN slightly reduced the prevalence of the high-frequency clusters, with the high-frequency cluster characterized by high levels of frequency modulation being virtually absent in *Tph2*^*−/−*^ pups exposed to CN (Fig. 5G-L).

**Fig. 6:**
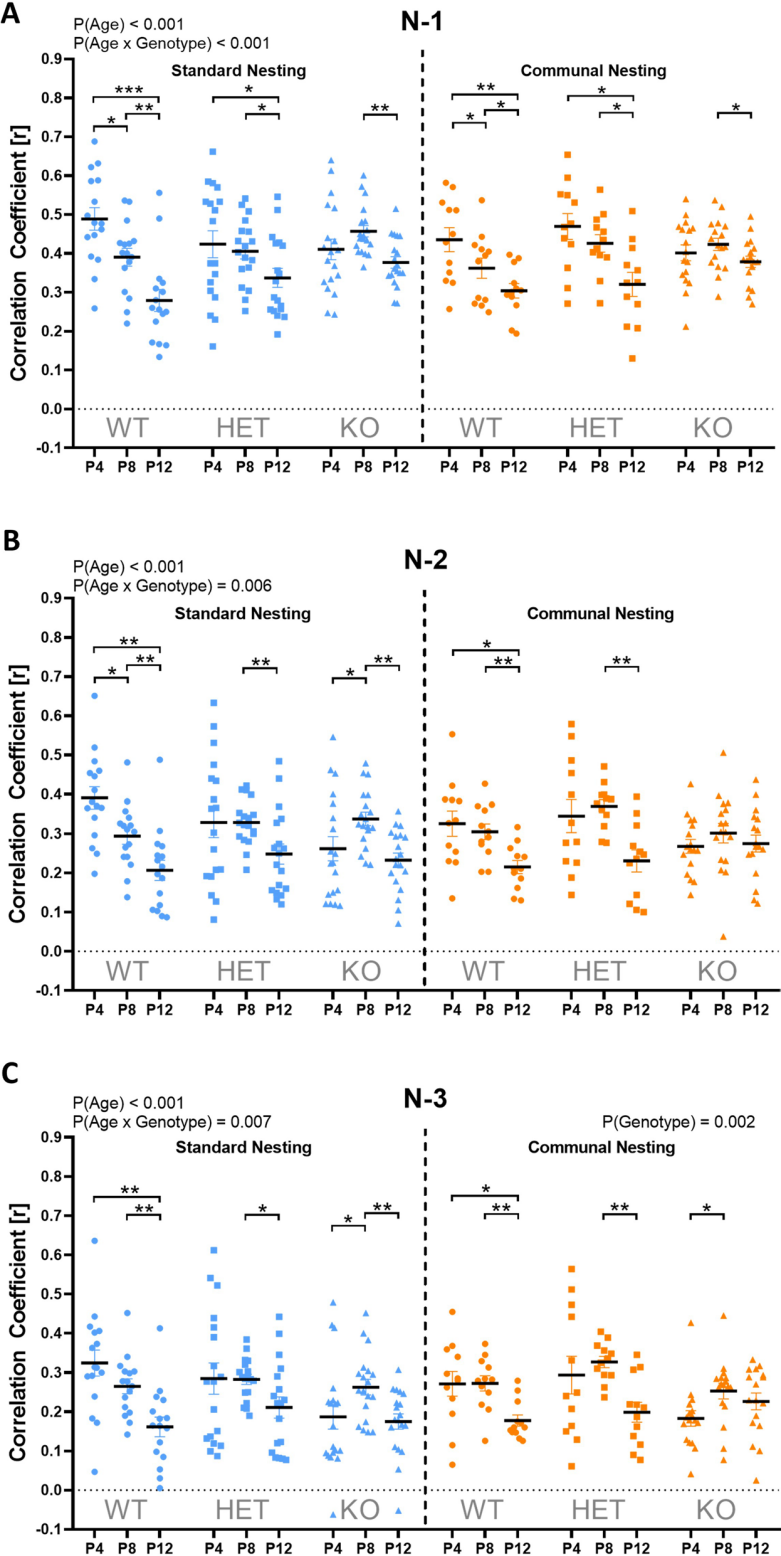
*Tph2* deficiency in rats affects the temporal organization of the emission of isolation-induced ultrasonic vocalizations. (**A-C**) Correlation coefficients (r) between call durations reflecting the sequential organization of isolation-induced ultrasonic vocalizations in *Tph2*^*−/−*^ (KO) and *Tph2*^*+/−*^ (HET) rat pups, compared to *Tph2*^*+/+*^ (WT) littermate controls. Correlation coefficients (r) indicate the level of correlations between the durations of a given isolation-induced ultrasonic vocalizations with the call durations of the previous ones (**A**, N-1), the ones two before (**B**, N-2), and the ones three before (**C**, N-3) on postnatal days (P) 4, 8, and 12. SN = standard nesting (blue), CN = communal nesting (orange). Data are presented as mean ± SEM. Effect of age: *** p < .001; ** p < .01: * p < .05

Finally, *Tph2* deficiency affected the temporal organization of isolation-induced USV emission. This is suggested by sequential analyses through correlating the call durations of given isolation-induced USV with the call durations of previous ones. In general, correlations between a given isolation-induced USV were highest with the previous one (N-1) and gradually declined when correlating a given isolation-induced USV with the one two before (N-2) or even three before (N-3). This pattern was evident in all genotypes. In *Tph2*^*+/+*^ littermate controls, however, such correlations decreased across developmental stages from P4 over P8 to P12, with higher variability at later stages possibly reflecting a higher level of temporal organization or complexity. This was seen for all three types of correlations (N-1, N-2, and N-3). Interestingly, no such change was seen in *Tph2*^*−/−*^ pups. There, the correlations remained mostly unchanged from P4 over P8 to P12, with evidence for a mild inverted U-shaped pattern. This indicates that the temporal call emission pattern remains highly stereotypic even at later developmental stages, with values on P12 being similar to P4, lacking of the increase in temporal organization or complexity seen in *Tph2*^*+/+*^ littermates. *Tph2*^*+/−*^ pups displayed an intermediate phenotype for N-1 (age × genotype: F_3.715,152.33_ = 5.838; p < 0.001; Fig. 6A), N-2 (age × genotype: F_3.358,137.698_ = 4.09; p = 0.006; Fig. 6B), and N-3 (age × genotype: F_3.171,129.992_ = 4.133; p = 0.007; Fig. 6C). Importantly, the highly stereotypic temporal call emission pattern displayed by *Tph2*^*−/−*^ pups was primarily seen under conditions of close temporal proximity (N-1 and N-2), while under conditions of low temporal proximity (N-3) correlations were lower than in *Tph2*^*+/+*^ littermates (genotype: F_2,82_ = 6.81; p = 0.002; Fig. 6C). Of note, similar results were obtained for SN and CN conditions, with developmental changes being slightly more prominent under SN conditions (not shown in detail).

Taken together, *Tph2* deficiency led to a prominent reduction in the emission of isolation-induced USV. Moreover, *Tph2* deficiency was associated with acoustically-altered isolation-induced USV characterized by untypically long call durations and high peak frequencies but low peak amplitudes together with low levels of frequency modulation. Call clustering and the temporal organization of isolation-induced USV were also affected by *Tph2* deficiency, with differences in the relative prevalence of call clusters being less prominent and the sequential call emission pattern being more stereotypic in *Tph2*-deficient rat pups. The effects of *Tph2* deficiency on isolation-induced USV were mostly not modulated by CN.

### Homing test as a proxy maternal affiliation

**Fig. 7:**
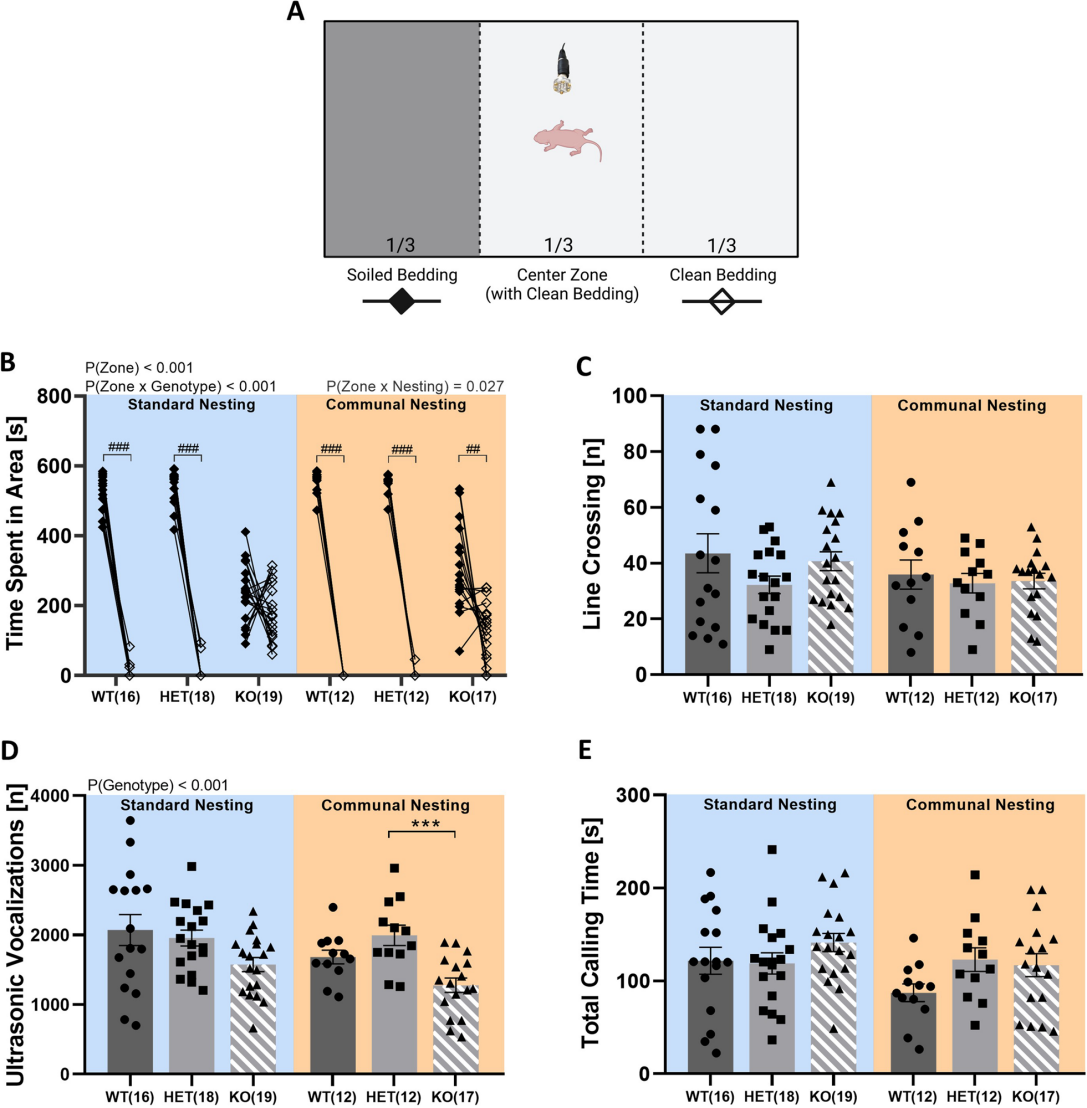
*Tph2* deficiency in rats leads to maternal affiliation deficits, which can be rescued through communal nesting (CN), as compared to standard nesting (SN). (**A**) Overview of the homing test as a proxy for maternal affiliation in in *Tph2*^*−/−*^ (KO) and *Tph2*^*+/−*^ (HET) rat pups, compared to *Tph2*^*+/+*^ (WT) littermate controls. (**B**) Maternal affiliation as measured through the time spent in the clean bedding zone versus the soiled bedding zone containing maternal odors, depending on nesting condition. (**C**) Total number of line crossings, depending on nesting condition. (**D**) Total number of isolation-induced ultrasonic vocalizations, depending on nesting condition. (**E**) Total calling time, depending on nesting condition. SN = standard nesting (blue), CN = communal nesting (orange). Data are expressed as mean ± SEM. Effect of genotype: *** p < .001; Effect of zone: ### p < .001, ## p < .01. N(WT-SN) = 16, N(HET-SN) = 18, N(KO-SN) = 19, N(WT-CN) = 12, N(HET-CN) = 12, N(KO-CN) = 17

*Tph2* deficiency in rats resulted in severely impaired maternal affiliation, as assessed by the pup’s preference for its mother’s odor in the homing test (Fig. 7A). This impairment was ameliorated by CN. As expected, the large majority of rat pups displayed a strong preference for the one third of the test apparatus with soiled bedding containing the mother’s odor over the one third of the test apparatus with clean bedding without the mother’s odor (zone: F_2,81_ = 890.722; p < 0.001; Fig. 7B). This preference was modulated by *Tph2* deficiency, with *Tph2*^*+/−*^ pups and *Tph2*^*+/+*^ littermate controls but not *Tph2*^*−/−*^ pups displaying strong preferences for the zone with soiled bedding containing the mother’s odor (genotype: F_2,81_ = 72.631; p < 0.001; zone ×  genotype: F_2,81_ = 130.372; p < 0.001). Importantly, this preference was affected by CN (zone × nesting: F_1,81_ = 5.052; p = 0.027; zone × nesting × genotype: F_2,81_ = 2.051; p = 0.135). Specifically, under SN conditions, *Tph2*^*+/−*^ pups and *Tph2*^*+/+*^ littermates displayed a prominent preference for soiled bedding, while no preference was seen in *Tph2*^*−/−*^ pups. While *Tph2*^*+/−*^ pups and *Tph2*^*+/+*^ littermates spent ~ 91% and ~ 88% of time on average, respectively, in the one third of the test apparatus with soiled bedding, *Tph2*^*−/−*^ pups spent only ~ 39% of time on average in soiled bedding, i.e. only slightly above chance level of 33%. CN exerted beneficial effects in enhancing maternal affiliation, as reflected in a stronger preference for soiled bedding. Beneficial effects of CN were primarily evident in *Tph2*^*−/−*^ pups, leading to ~ 51% of time spent in soiled bedding, i.e. clearly above chance level of 33%, reflecting a prominent increase of ~ 12%, as compared to SN. In contrast, the beneficial effects of CN on *Tph2*^*+/−*^ pups and *Tph2*^*+/+*^ littermates were mild, likely due to the strong preference already shown under SN conditions. *Tph2*^*+/−*^ pups and *Tph2*^*+/+*^ littermates spent ~ 92% and ~ 93% of time on average, respectively, in the one third of the test apparatus with soiled bedding, reflecting an increase of merely 1% and 5%, respectively, as compared to SN. Of note, no evidence for genotype differences were obtained for line crossing (genotype: F_2,82_ = 1.544; p = 0.220; nesting: F_1,82_ = 2.456; p = 0.121; nesting × genotype: F_2,82_ = 0.506; p = 0.605), suggesting that the maternal affiliation deficits displayed by *Tph2*^*−/−*^ pups were not due to locomotor impairment (Fig. 7C).

Consistent with the reduction in isolation-induced USV, USV emission rates during the homing test were affected by genotype. *Tph2*^*−/−*^ pups again displayed a reduced rate of isolation-induced USV emission, as compared to *Tph2*^*+/−*^ pups and *Tph2*^*+/+*^ littermate controls (genotype: F_2,82_ = 9.915; p < 0.001; nesting × genotype: F_2,82_ = 1.334; p = 0.269; Fig. 7D), suggesting a robust phenotype independent of test context. However, total calling time was found to be comparable to the levels seen in *Tph2*^*+/−*^ pups and *Tph2*^*+/+*^ littermates (genotype: F_2,82_ = 1.943; p = 0.150; nesting × genotype: F_2,82_ = 1.297; p = 0.279; Fig. 7E). This may indicate that the homing test environment caused alterations in acoustic features, as compared to the test environment used for studying the developmental trajectories of isolation-induced USV.

Taken together, *Tph2* deficiency led to severely impaired maternal affiliation, yet this impairment was ameliorated by CN.

### Maternal preference test

**Fig. 8:**
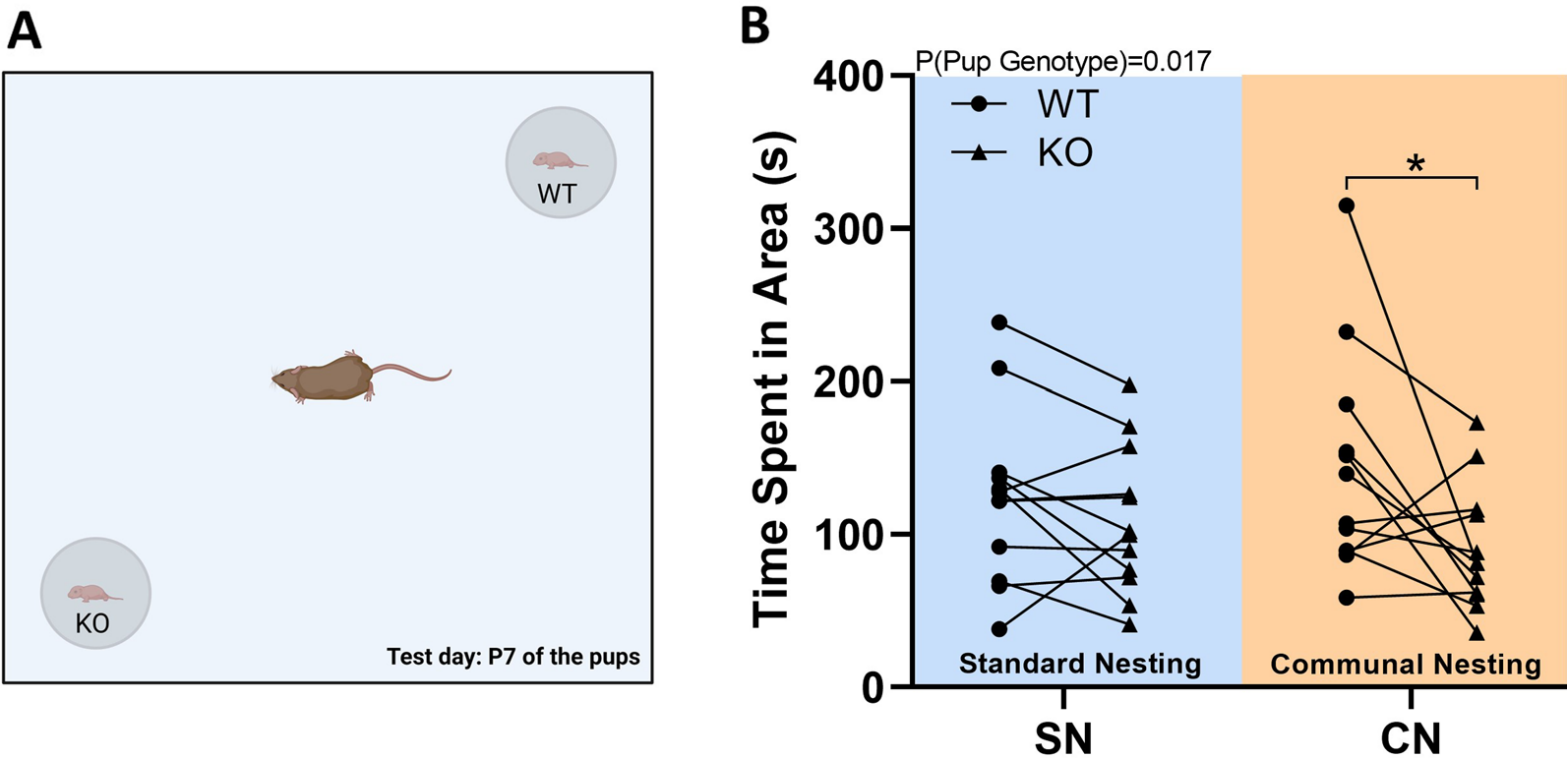
*Tph2* deficiency in rats causes a reduction in maternal preference, which is emphasized through communal nesting (CN), as compared to standard nesting (SN). (**A**) Overview of the maternal preference test to close the communicative loop between mother and pup and to compare maternal preferences between *Tph2*^*−/−*^ (KO) rat pups and *Tph2*^*+/+*^ (WT) littermate controls. (**B**) Time spent by the mother in proximity to WT versus KO rat pups. Of note, the individual data points of an individual WT rat pup and an individual KO rat pup are connected by a line in order to indicate that the two connected rat pups were simultaneously exposed to their mother. Mothers were always exposed to their own pups. SN = standard nesting (blue), CN = communal nesting (orange). Data are expressed as mean ± SEM. Effect of pup genotype: *p < .05. N(SN) = 12 WT-KO pairs, N(CN) = 12 WT-KO pairs

In order to close the communicative loop between mother and pup, maternal preference was assessed by exposing one *Tph2*^*+/+*^ littermate control and one *Tph2*^*−/−*^ pup simultaneously to their mother (Fig. 8A). Maternal preference was depending on the genotype of the pup, with mothers showing a preference for *Tph2*^*+/+*^ littermate controls over *Tph2*^*−/−*^ pups (pup genotype: F_1,22_ = 6.721; p = 0.017; Fig. 8B). While CN had no prominent modulatory effect (nesting: F_1,22_ < 0.001; p = 0.995; nesting × pup genotype: F_1,22_ = 2.059; p = 0.165), it is worth noting that the mothers displayed a more prominent preference for *Tph2*^*+/+*^ littermates over *Tph2*^*−/−*^ pups under CN conditions (t_11_ = 2.239; p = 0.047) than under SN conditions (t_11_ = 1.323; p = 0.213).

Taken together, *Tph2* deficiency led to a reduced ability to attract the mother, particularly under CN.

## Discussion

A lack of 5-HT in the brain due to deficiency of the rate-limiting enzyme in 5-HT synthesis, TPH2, was recently reported to result in impaired maternal affiliation across species, including mice, rats, and monkeys [47]. In rodents, this was reflected in a lack of preference for maternal odors and reduced levels of isolation-induced USV, possibly contributing to a severe growth retardation phenotype. Here, we tested whether growth retardation, maternal affiliation deficits, and/or impairments in socio-affective communication caused by *Tph2* deficiency can be rescued through early social enrichment in rats. To this aim, we compared male and female *Tph2*^*−/−*^ knockout and *Tph2*^*+/−*^ heterozygous rat pups to *Tph2*^*+/+*^ wildtype littermate controls, with litters being randomly assigned to SN (one mother with her litter) or CN (two mothers with their two litters).

One of the most prominent effects of *Tph2* deficiency is growth retardation. Growth retardation is seen in mice [2], rats [37], and pigs [44]. The growth retardation phenotype was evident during the first two weeks of life in the present study. While body weight increased substantially in *Tph2*^*+/−*^ pups and *Tph2*^*+/+*^ littermate controls, body weight gain was reduced in *Tph2*^*−/−*^ pups. The measures obtained in the present study indicate that first differences in body weight are detectable from postnatal day 4. It is possible that the reduced body weight gain in *Tph2*^*−/−*^ pups was caused by reduced feeding behavior. In fact, milk spots in the belly were less visible in *Tph2*^*−/−*^ pups during the first week of life, as compared to *Tph2*^*+/−*^ pups and *Tph2*^*+/+*^ littermates, possibly reflecting dys-/hypophagia due to reduced suckling activity. Surprisingly, the growth retardation phenotype displayed by *Tph2*^*−/−*^ pups was aggravated by CN, as reflected in even further reduced body weight gain in *Tph2*^*−/−*^ pups exposed to CN, as compared to SN. This is surprising because CN was repeatedly shown to promote growth in mice [32], even when the ratio of mothers to offspring was the same as in SN [66], an effect also seen in *Tph2*^*+/−*^ pups and *Tph2*^*+/+*^ littermates in the present study. However, in rats there is also evidence suggesting that CN can result in lower body weight gain [51], possibly due to increased competition between pups, particularly when pups differ in body size [49]. It thus appears possible that the presence of a second litter during CN conditions made it even more difficult for *Tph2*^*−/−*^ pups to gain access to the mother for milk. While previous studies suggested that growth retardation is not linked to altered food intake in *Tph2*-deficient mice because of filled milk pouches [59], aggravation of the growth retardation phenotype might be caused by the more competitive conditions of CN that limit suckling behavior. Irrespective of CN, the growth retardation phenotype was associated with a delay in eye opening, consistent with a previous study in mice [38].

Growth retardation in *Tph2*^*−/−*^ pups was further associated with impairments in thermoregulatory capabilities, as reflected in lower body temperature values in *Tph2*^*−/−*^ pups than in *Tph2*^*+/−*^ pups and *Tph2*^*+/+*^ littermate controls. While the developmental pattern was similar to body weight gain, effects of *Tph2* deficiency on thermoregulatory capabilities were not aggravated by CN. Improved thermoregulatory capabilities with age were seen irrespective of genotype. As no delay in fur development was detected, it appears likely that the impairments in thermoregulatory capabilities are not the consequence of a delay in development but associated with the lack of 5-HT in the brain. This is consistent with a prominent role of the 5-HT system in regulating body temperature [53]. In fact, *Tph2* deficiency was repeatedly shown to cause impairments in thermoregulatory capabilities in mice [2] and rats [37]. However, impaired thermoregulation does not appear to drive growth retardation in *Tph2*-deficient mice, as increasing nest temperature did not ameliorate the growth retardation phenotype [59].

Moreover, *Tph2*^*−/−*^ pups displayed a broad variety of alterations in somatosensory reflexes, as compared to *Tph2*^*+/−*^ pups and *Tph2*^*+/+*^ littermate controls, irrespective of CN. Interestingly, *Tph2*^*−/−*^ pups displayed improved performance in a few of the assays used for quantifying somatosensory reflexes. This includes grasping, righting, and vertical screen holding. Possibly, this is due to their lower body weight. For example, lower body weight might have made it easier for them to flip over onto the abdomen during righting reflex assessment. Likewise, lower body weight might have helped them to stay longer on a square grid at 90° angle. However, reduced performance was seen in most of the assays, including forelimb placing, level screen, negative geotaxis, cliff avoidance, and bar holding. In line with a previous report in mice [38], the impairment in negative geotaxis was particularly prominent, and could amongst others reflect impairments in vestibular sensation. Together, this indicates that CN aggravates the growth retardation phenotype caused by *Tph2* deficiency but does not modulate its effects on developmental milestones, somatosensory reflexes, and thermoregulatory abilities in a prominent manner.

*Tph2* deficiency in rats further led to severe deficits in socio-affective communication during the first two weeks of life, as evidenced by reduced emission rates of isolation-induced USV. While *Tph2*^*+/−*^ pups and *Tph2*^*+/+*^ littermate controls emitted about 1500–1600 isolation-induced USV on average, *Tph2*^*−/−*^ pups emitted only about 800–900 isolation-induced USV. This strong reduction is in line with a rich variety of evidence supporting the notion that the 5-HT system plays a prominent role in regulating socio-affective communication in mice and rats [87].

Specifically, the present findings of reduced emission rates in rat pups lacking 5-HT in the brain due to *Tph2* deficiency are in line with a large body of research on pup ultrasonic calling [60]. Notably, this includes the first study on the role of 5-HT in regulating isolation-induced USV in rat pups conducted by Hård et al. (1982). In this study, they demonstrated that lesioning central 5-HT neurons through administering the neurotoxin 5,7-dihydroxytryptamine (5,7-DHT) blocks pup ultrasonic calling. Consistent with the present findings, they showed that inhibiting 5-HT synthesis by para-chlorophenylalanine (PCPA) led to a clear reduction in pup ultrasonic calling; a finding recently replicated [47]. Importantly, the effects of the partial and temporal 5-HT depletion caused by PCPA were dose-dependently reversed by administering the precursor 5-hydroxytryptophan (5-HTP), indicating that the acute deficiency in 5-HT availability caused the reduction in pup ultrasonic calling [30]. Following the pioneering study by Hård et al. [30], specific components of the complex 5-HT system were selectively targeted to better understand their individual contributions in regulating the emission of isolation-induced USV. This included 5-HT synthesis, breakdown, and reuptake mechanisms, together with the functions of autoreceptors and postsynaptic receptors. Together, they confirm an important role of 5-HT in regulating the emission of isolation-induced USV in rat pups. For example, inhibiting 5-HT function through the application of 8-OH-DPAT, which works mostly through activation of the 5-HT1A autoreceptor, consistently decreased pup ultrasonic calling in a dose-dependent manner [29, 35, 36, 42, 77, 79]. Likewise, partial 5-HT1A agonists, such as buspirone, consistently and dose-dependently decreased isolation-induced USV in rat pups [16, 33, 34, 40–42, 77, 79]. Moreover, a wide range of selective 5-HT reuptake inhibitors, such as escitalopram, fluoxetine, and paroxetine, were found to reduce pup ultrasonic calling [33, 34, 43, 72, 78, 89].

Importantly, the reduction in isolation-induced USV observed in rat pups devoid of brain 5-HT due to *Tph2* deficiency is also consistent with a study on *Tph2*-deficient mouse pups [54]. In this study, *Tph2*-deficient mouse pups displayed a clear deficit in the emission of isolation-induced USV, particularly during the first week of life; a finding recently replicated and shown to be robust under room temperature [47]. While the emission rates in wildtype littermate controls were characterized by the for mice typical inverted U-shaped developmental emission pattern, this pattern was weaker in *Tph2*-deficient mouse pups [54]. Evidence for altered developmental trajectories were also obtained in the present study, yet strongest deficits were seen during the second week of life. While isolation-induced USV emission rates were more than doubled in *Tph2*^*+/−*^ pups and *Tph2*^*+/+*^ littermates across development, there was no such prominent increase seen in *Tph2*^*−/−*^ pups. The fact that the effects of *Tph2* deficiency on pup ultrasonic calling were seen later in rats than mice might be due to differences in the developmental profiles of the two species. Notably, reduced levels of isolation-induced USV were recently also reported in a comparative study including rats, where it was found that the deficits displayed by *Tph2*-deficient rats cannot be rescued by oxytocin treatment [47].

The deficits in socio-affective communication driven by *Tph2* deficiency in rats were also reflected in alterations of acoustic features of USV emitted by *Tph2*^*−/−*^ pups. This included all four main acoustic features, i.e. call duration, peak frequency, peak amplitude, and frequency modulation. Alterations in acoustic features were often particularly strong during the second week of life. As compared to *Tph2*^*+/−*^ pups and *Tph2*^*+/+*^ littermate controls*, Tph2*^*−/−*^ pups emitted isolation-induced USV that were slightly longer and characterized by higher peak frequency but lower peak amplitude and frequency modulation. In mice, *Tph2* deficiency was associated with isolation-induced USV characterized by lower peak frequency and peak amplitude [54]. Moreover, call clustering was affected.

In the present study, density plots integrating the information obtained through detailed spectrographic analyses of more than 150,000 individual isolation-induced USV revealed multiple clusters of call subtypes. When plotting peak frequency against call duration two call clusters were revealed in *Tph2*^*+/+*^ littermate controls. One prominent cluster was characterized by relatively low peak frequencies roughly between 40 and 50 kHz. A second cluster was characterized by relatively high peak frequencies roughly between 60 and 70 kHz. For both clusters, two call subtypes were present, differing in frequency modulation, i.e. weakly versus strongly frequency-modulated. Evidence for similar call clustering was obtained in a strain comparison study on isolation-induced USV, where three call subtypes were identified in Long-Evans, Sprague–Dawley, and Wistar rat pups [68]. As in the present study using Dark Agouti rats, the low-frequency cluster characterized by low levels of frequency modulation was most prevalent. The high-frequency cluster characterized by high levels of frequency modulation that was least prevalent in the present study was not detected in the strain comparison study, possibly due to the lower number of isolation-induced USV included in the analysis. The consistency in call clustering across strains is remarkable given the different analytical approaches and the low level of relatedness between Dark Agouti rats on the one side and Long-Evans, Sprague–Dawley, and Wistar rats on the other side.

Although all four call clusters were present in *Tph2*^*−/−*^ pups, there were two prominent genotype differences in prevalence. Firstly, in *Tph2*^*+/+*^ littermate controls the low-frequency cluster was much more prominent than the high-frequency cluster and this difference in relative prevalence was smaller in *Tph2*^*−/−*^ pups due to a more prominent high-frequency cluster. Secondly, the two call clusters characterized by high levels of frequency modulation were less prevalent in *Tph2*^*−/−*^ pups than in *Tph2*^*+/+*^ littermates. In particular, the high-frequency cluster characterized by high levels of frequency modulation was almost absent. Together, this indicates that the increased prevalence of the high-frequency cluster in *Tph2*^*−/−*^ pups compared to *Tph2*^*+/+*^ littermates was exclusively driven by isolation-induced USV characterized by low levels of frequency modulation. Besides prevalence, *Tph2* deficiency affected the typical peak frequency associated with each of the four clusters. For all four clusters, peak frequency was shifted up by about 5 kHz. Alterations in call clustering were also reported in *Tph2*-deficient mice, with less clearly segregated clusters as the most prominent feature [54]. Interestingly, changes in call clustering were observed in a number of genetic mouse models displaying behavioral alterations with relevance to neurodevelopmental disorders. This includes the BTBR T + tf/J mouse model for ASD [81], *Shank1*-deficient mice [73], *Myt1l*-deficient mice [83], and mice lacking the cannabinoid receptor 1 [24] or the cell adhesion molecule protocadherin10 selectively in interneurons [1].

Besides call clustering, *Tph2* deficiency affected the temporal organization of isolation-induced USV emission. This is suggested by sequential analyses through correlating the call durations of given isolation-induced USV with the call durations of previous ones, as a proxy for the level of randomness versus stereotypy in the temporal USV emission patterns [80]. In *Tph2*^*+/+*^ littermate controls, such correlations decreased across developmental stages during the first weeks of life, with higher variability at later stages possibly reflecting a higher level of temporal organization or complexity. Interestingly, no such change was seen in *Tph2*^*−/−*^ pups. There, the correlations remained unchanged across development. This indicates that the temporal call emission pattern remains highly stereotypic even at later developmental stages, with values on postnatal day 12 being similar to postnatal day 4, lacking the increase in temporal organization or complexity seen in *Tph2*^*+/+*^ littermates. While there is little known about the temporal organization of isolation-induced USV emission in rat pups, evidence for a non-random temporal organization was obtained in mice before, using call durations [80] or intervals between calls [23] as a proxy. As in rat pups, the temporal organization is decreasing with development in mice [73]. Interestingly, alterations in the temporal organization were also found in *Tph2*^*−/−*^ mouse pups, yet changes observed in mice were indicative of a more random emission pattern [54].

CN did not affect the emission of isolation-induced USV in a prominent manner and no robust evidence was obtained for a rescue of the socio-affective communication deficits caused by *Tph2* deficiency. In fact, the strong reduction in isolation-induced USV of about 50% in *Tph2*^*−/−*^ pups was also seen under CN conditions. Whereas isolation-induced USV emission rates ranged between about 1500–1600 in *Tph2*^*+/−*^ pups and *Tph2*^*+/+*^ littermate controls under SN and CN conditions, CN did not ameliorate the socio-affective communication deficit displayed by *Tph2*^*−/−*^ pups and their emission rate remained around 800–900 isolation-induced USV. There is little known about the effects of CN on pup ultrasonic calling. A recent study in rats reported CN effects, yet comparisons are difficult to make because rats selectively bred for low or high rates of isolation-induced USV were used [51]. In this study, it was reported that CN inhibits pup ultrasonic calling in the high but not the low rat line. However, no unselected control line was included. A modulatory effect of CN was also reported in mice [17]. To our knowledge, the present study is the first to reveal effects of CN on acoustic features, call clustering, and temporal organization. Irrespective of genotype, CN led to minor alterations in the acoustic features, most notably reductions in peak frequency and frequency modulation. Moreover, CN slightly reduced the prevalence of the high-frequency cluster, with the high-frequency cluster characterized by high levels of frequency modulation being virtually absent in *Tph2*^*−/−*^ pups exposed to CN. Of note, similar temporal organization results were obtained for SN and CN conditions, with developmental changes being slightly more prominent under SN conditions.

Although CN did not rescue the impairments in socio-affective communication, CN ameliorated the maternal affiliation deficit caused by *Tph2* deficiency. Recently, it was reported that *Tph2* deficiency results in impaired maternal affiliation across species [47]. This includes mice, rats, and monkeys. In mice and rats, maternal affiliation deficits were reflected in a lack of preference for maternal odors in the homing test. In monkeys, reduced maternal affiliation was indicated by longer approach latencies towards the anesthetized mother and a lack of preference for pictures of their own mother over another mother. Consistent with those observations [47] and previous ones in mice [38], *Tph2* deficiency in rats resulted in severely impaired maternal affiliation in the present study. As in the other studies, the pup’s preference for its mother’s odor in the homing test was used as a proxy for maternal affiliation. As expected, most rat pups displayed a strong preference for the one third of the test apparatus with soiled bedding containing the mother’s odor over the one third of the test apparatus with clean bedding without the mother’s odor. This preference was modulated by *Tph2* deficiency. Under SN conditions, *Tph2*^*+/−*^ pups and *Tph2*^*+/+*^ littermate controls but not *Tph2*^*−/−*^ pups displayed strong preferences for the zone with soiled bedding containing the mother’s odor. CN rescued this phenotype and exerted beneficial effects in enhancing maternal affiliation, as reflected in a stronger preference for soiled bedding. While under SN condition *Tph2*^*−/−*^ pups spent only about 39% of their time in soiled bedding, they spent there about 51% of their time under CN conditions, i.e. clearly above chance level of 33%.

Consistent with the reduction in isolation-induced USV across developmental stages, pup ultrasonic calling during the homing test was affected by genotype. *Tph2*^*−/−*^ pups again displayed reduced pup ultrasonic calling, as compared to *Tph2*^*+/−*^ pups and *Tph2*^*+/+*^ littermate controls, suggesting a robust phenotype independent of test context. This appears to be inconsistent with the findings obtained by Liu et al. [47]. In their study, deficits in pup ultrasonic calling were exclusively seen under clean bedding conditions but not in mice or rats exposed to bedding containing odor from the mother or an unfamiliar male. While Liu et al. [47] recorded isolation-induced USV in separate test contexts differing in the presence of social cues, we recorded during the homing test. The discrepancy may therefore be attributed to the complexity of the homing test, which contains both clean and soiled bedding containing maternal odor, a configuration likely to affect pup ultrasonic calling. Notably, compared to *Tph2*^*+/−*^ pups and *Tph2*^*+/+*^ littermates, which spent most of their time in soiled bedding, *Tph2*^*−/−*^ pups might have been affected more by the complexity of the environment, as they spent much less time in the soiled bedding. It thus appears promising to perform an in-depth analysis of pup ultrasonic calling time-synced to the behavioral profile, i.e. to the position of the pup in the homing test, comparing clean and soiled bedding time windows.

To our knowledge, this is the first study providing evidence for a positive effect of CN on social affiliation during early developmental stage in rodents. Liu et al. [47] demonstrated that administering the precursor 5-HTP or oxytocin can rescue the maternal affiliation phenotype in *Tph2*-deficient rat pups, and it is interesting to note that oxytocin treatment did rescue the maternal affiliation deficit but not the deficit in pup ultrasonic calling [47], mirroring the present result pattern associated with CN. It is thus tempting to speculate that CN exerts positive effects through changes in the oxytocin system, which was found to be altered in *Tph2*-deficient rats [52]. It was previously shown that CN can enhance oxytocin receptor levels in the amygdala in mice, potentially contributing to the beneficial effects of CN on social behavior [13, 14]. In fact, CN leads to prominent changes particularly in the social domain. This is reflected in enhanced levels of social interaction behavior, such as rough-and-tumble play in rats [51], as well as increased social competence in establishing social hierarchies in mice [19]. Moreover, there is evidence for more efficient social recognition processes in mice following CN, as indicated by the emission of affiliative USV [25].

The maternal affiliation deficit displayed by *Tph2*^*−/−*^ pups might be associated with the growth retardation phenotype. It seems plausible to assume that reduced maternal affiliation behavior can result in reduced feeding behavior not compensated by maternal care. Given that CN ameliorates maternal affiliation deficits, however, one would expect the growth retardation phenotype to be ameliorated and not to be aggravated under CN conditions. It therefore appears possible that the impairment in socio-affective communication plays a prominent role in the growth retardation phenotype. This view is supported by previous observations suggesting that genotype-dependent differences in body weight cannot be detected during prenatal development but only emerge slowly after birth [59]. In fact, in the present study first evidence of a growth retardation phenotype was obtained around the time when genotype differences in the emission of isolation-induced USV were detected the first time. A similar pattern was obtained in mice, where *Tph2*-deficient mouse pups displayed the deficit in isolation-induced USV exactly during growth retardation onset [54]. It is thus possible that the deficits in isolation-induced USV production displayed by *Tph2*^*−/−*^ pups contribute to their growth retardation phenotype.

In fact, pup ultrasonic calling serves important communicative functions in regulating mother–pup interactions and was shown to stimulate maternal caregiving behavior in mice [69, 70] and rats [71, 85]. It thus appears likely that the few and acoustically-altered isolation-induced USV emitted by *Tph2*^*−/−*^ pups are less efficient in attracting mothers. Impaired socio-affective communication in *Tph2*^*−/−*^ pups might therefore explain the growth retardation phenotype emerging during early postnatal development. To test that idea, we decided to close the communicative loop between mother and pup and assessed maternal preferences by exposing mothers to one *Tph2*^*+/+*^ littermate control and one *Tph2*^*−/−*^ pup simultaneously. This showed that maternal preferences were depending on the genotype of the pup, with mothers showing a preference for *Tph2*^*+/+*^ littermate controls over *Tph2*^*−/−*^ pups. Interestingly, mothers displayed a clear preference for *Tph2*^*+/+*^ littermate controls over *Tph2*^*−/−*^ pups primarily under CN conditions. Although it is currently unclear why a stronger preference for *Tph2*^*+/+*^ littermate controls is seen under CN conditions, this stronger preference is consistent with the aggravated growth phenotype in *Tph2*^*−/−*^ pups exposed to the more competitive CN environment.

It was previously shown that acoustic features of isolation-induced USV determine at least partially how efficient they are in eliciting maternal search and retrieval behavior. Through systematically manipulating acoustic features in mice, evidence for a categorical perception mechanism was obtained and it was shown that isolation-induced USV falling into a specific frequency range are more efficient than others [20–22]. Although no systematic studies on the effects of acoustic features, call clustering, and temporal organization on search and retrieval behavior in rats are available to our knowledge, it appears likely that not only the reduction in isolation-induced USV emission rates but also those more subtle changes contributed to the reduced preference mothers displayed for *Tph2*^*−/−*^ pups. That maternal preferences displayed during a maternal preference task similar to the one applied in the present study can be related to differences in pup ultrasonic calling was demonstrated previously. For example, a study on two substrains of mice, C57BL/6JOlaHsd and C57BL/6NCrl, reported that C57BL/6JOlaHsd mouse pups emitting more isolation-induced USV characterized by higher peak amplitude were more efficient in attracting the mother [82]. This finding aligns well with the present results where *Tph2*^*−/−*^ pups emitting isolation-induced USV with lower peak amplitude starting from postnatal day 6 received less attention of their mother on postnatal day 7. Of note, however, there is also contradictory evidence where mothers preferred mutant pups emitting fewer isolation-induced USV over wildtype littermate controls, yet detailed information about acoustic features is lacking [11]. Together, this supports the view that impairments in socio-affective communication between mother and pup contribute to the growth retardation phenotype. This is in line with a cross-fostering study in *Tph2*-deficient mice, where it was shown that both, the genotype of the pup and the genotype of the mother, determine at least partially maternal caregiving behavior [8].

## Limitations

While our study has a number of strengths, such as a systematic and comprehensive comparison of CN effects, detailed spectrographic analysis of isolation-induced USV across development, testing of male and female rat pups, and the inclusion of a maternal preference test to truly model the reciprocal nature of communication, our study has limitations as well. Firstly, we applied an intensive health monitoring and care system. To mitigate for the low survival rates of only about 50% previously reported to be associated with *Tph2* deficiency in mice [2] and rats [37], we performed health monitoring, provided agar food, and removed over-represented genotypes to increase maternal care per pup through reducing litter size. These measures and the resulting very high survival rate of about 90% make it more difficult to compare our results with previous studies. That said, we wish to emphasize that key aspects of the behavioral phenotype observed in the present study, such as the reduced levels of isolation-induced USV and the deficit in maternal affiliation behavior, are consistent with other reports, even across species [47, 54]. Secondly, it remains unclear how *Tph2*-deficiency leads to socio-affective communication deficits. One possibility is that *Tph2*-deficiency is associated with impairments in muscular control of isolation-induced USV production. Although the apparently improved performance in some of the somatosensory reflexes assessing muscular control, most notably vertical screen holding, speaks against a general muscular impairment, a study on muscular control of isolation-induced USV production might provide new insights. Thirdly, while the rescuing effect of CN is interesting, mechanistic insight as to why maternal affiliation deficits but not impairments in socio-affective communication can be rescued is needed. The findings on the oxytocin system by Liu et al. [47] provide an interesting entry point to relevant neurobiological mechanisms.

## Conclusion

Our findings show that CN aggravates growth retardation despite ameliorating maternal affiliation deficits in *Tph2*-deficient rat pups, possibly due to fewer and acoustically-altered isolation-induced USV, hindering efficient socio-affective communication between mother and pup. Our study is highlighting the need of gaining a better understanding of the complexity associated with socio-affective communication regulating mother–pup interactions through applying a truly communicative approach, including both, sender and receiver.

## Supplementary Information

Additional file 1.

## Data Availability

No datasets were generated or analysed during the current study.
