# Supplementary material for: Socio-affective communication in Tph2-deficient rat pups: communal nesting aggravates growth retardation despite ameliorating maternal affiliation deficits
Source: Mol Autism. 2024 Nov 29;15:50. doi: 10.1186/s13229-024-00629-x (PMC11606121; doi:10.1186/s13229-024-00629-x)
Supplement: Supplementary file 1 — Additional file 1. [file 13229_2024_629_MOESM1_ESM.pdf]

Supplementary Figure 1:

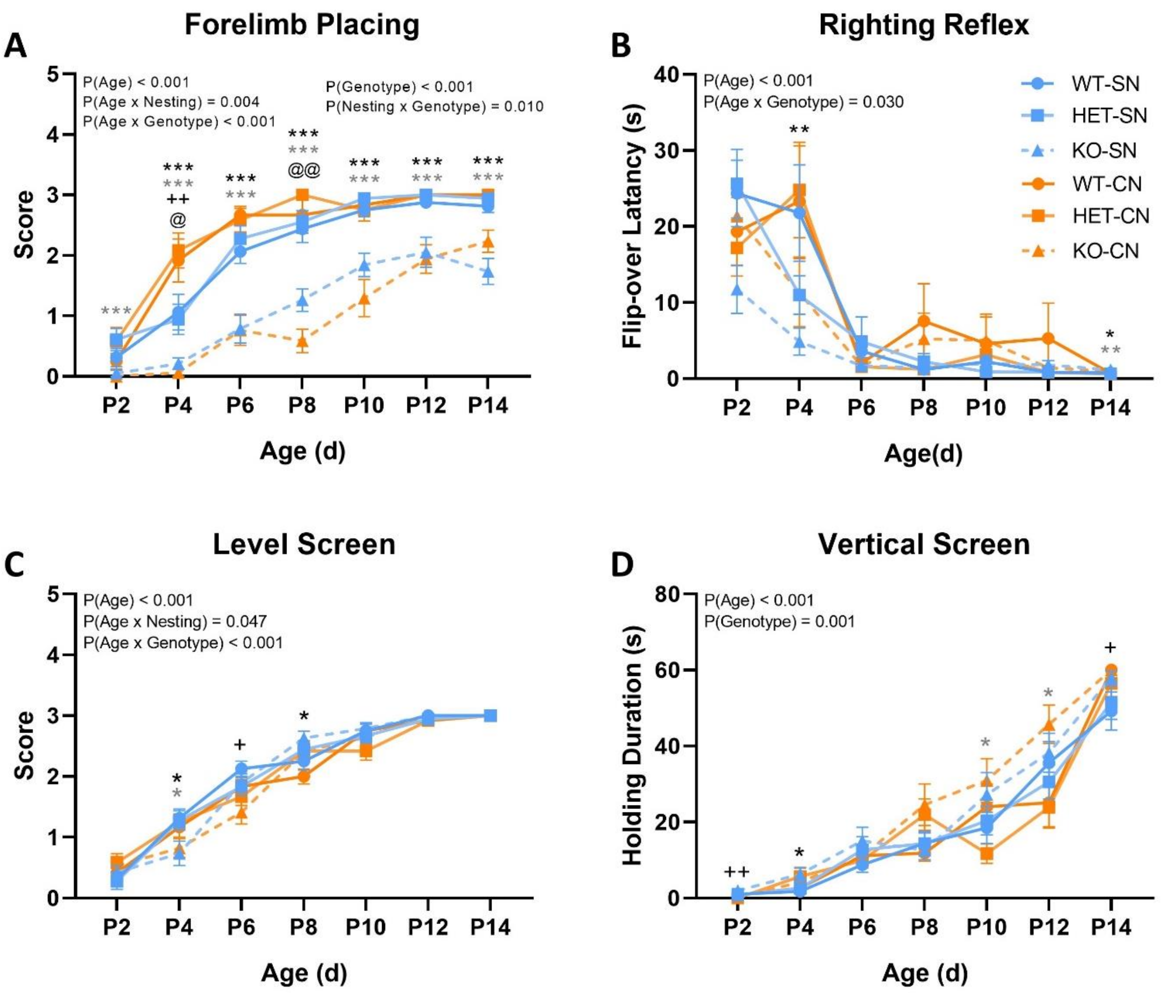

Supplementary Figure 1: *Tph2* deficiency in rats causes moderate impairments in somatosensory reflexes not modulated by communal nesting (CN), as compared to standard nesting (SN). (**A**) Score in the forelimb placing assay for WT, HET, and KO rat pups across P2 to P14, depending on nesting condition. (**B**) Flip-over latency in the righting reflex assay for WT, HET, and KO rat pups across P2 to P14, depending on nesting condition. (**C**) Score in the level screen assay for WT, HET, and KO rat pups across P2 to P14, depending on nesting condition. (**D**) Holding duration in the vertical screen assay for WT, HET, and KO rat pups across P2 to P14, depending on nesting condition. SN = standard nesting (blue), CN = communal nesting (orange). P = postnatal day. Data are expressed as mean±SEM. Effect of genotype: ***$^{(black)}$ WT vs. KO p<.001; **$^{(black)}$ WT vs. KO p<.01; *$^{(black)}$ WT vs. KO p<.05. ***$^{(gray)}$ HET vs. KO p<.001; **$^{(gray)}$ HET vs. KO p<.01; *$^{(gray)}$ HET vs. KO p<.05. Interaction nesting x genotype: @@ p<0.01, @ p<.05 . Effect of nesting: ++ p<.01; +p<.05.
